# Potential mutagenicity of aflatoxin B1 in Egyptian spices

**DOI:** 10.1186/s12864-024-11154-9

**Published:** 2025-01-06

**Authors:** Basma El Geoshi, Gehan El-Akabawy, Mohammed El Metwally, Magda I. Soliman

**Affiliations:** 1 Mansoura Medical Research Center (MERC), Faculty of Medicine, Mansoura University, Mansoura, Egypt; 2 Department of Basic Medical Sciences, College of Medicine, Centre of Medical and Bioallied Health Sciences Research, Ajman University, Ajman, United Arab Emirates; 3 Department of Anatomy and Embryology, Faculty of Medicine, Menoufia University, Menoufia, Egypt; 4 Mycological Research Department, Plant Pathology Research Institute, ARC, Giza, Egypt; 5 Botany Department, Faculty of Science, Mansoura University, Mansoura, Egypt

**Keywords:** Aflatoxin B1, RAPD, ISSR, GTS, Flow cytometry

## Abstract

The current study aimed to detect the mutagenic impacts of aflatoxin B1 (AFB1), which is produced by *Aspergillus* group fungi, via a high-plant genotoxicity test. Different durations of treatment (3 h, 6 h, and 12 h) were used to treat the *Vicia faba* root tips with varying concentrations of Aflatoxin B1 (AFB1) following the approved protocol for plant assays published by the International Program on Chemical Safety (IPCS) and the World Health Organization (WHO). The data obtained indicated that AFB1 not only has the ability to induce various alterations in the process of mitosis, ranging from increasing to decreasing mitotic and phase indices but also leads to many mitotic aberrations. The abnormalities observed varied on the basis of the ratio of AFB1 to treatment time. The aberrations included micronuclei in interphase, stickiness; two groups ring star disturbed and oblique metaphase late separation diagonal bridge and laggard and disturbed. anaphase and telophase. This study showed that biomonitoring *Vicia faba* is a sustainable method for estimating the cytotoxicity and genotoxicity of applied AFB1. Additionally, AFB1 caused changes in the protein profile detected by SDS‒PAGE, with each treated sample displaying a unique electrophoretic pattern due to the formation and disappearance of certain bands. The ISSR and RAPD assays changes in band numbers in all samples compared with the untreated control, and a decrease in genetic template stability (GTS) ratios was observed with higher levels of AFB1. The image cytometric data revealed a correlation between the dosage of AFB1 and its impact on cell cycle components in the meristematic cells of *Vicia faba* roots. Furthermore, an increase in AFB1 concentrationled to a decrease in B-cell lymphoma 2 (Bcl2) levels, an increase in chromatin condensation levels, and an increase in poly ADP‒ribose polymorphism (PARP) levels.

## Introduction

Aflatoxins are a class of hazardous secondary metabolites largely synthesized by closely related species belonging to the genus *Aspergillus* within the *Flavi* division [1] *Aspergillus* section *Flavi* includes 33 species as *A. flavus* and *A. parasiticus* are the major producers of aflatoxins, whereby the *A. flavus* produce B-series aflatoxins, while *A. parasiticus* produce both B- and G-series [2]. *Aspergilli* are widely distributed fungi globally and can synthesize Aflatoxins (Afs) in a diverse range of agricultural goods [2, 3]. The ingestion of Afs has been found to result in a diverse array of negative consequences, such as carcinogenicity, mutagenicity, teratogenicity, and immunosuppression [4]. Aflatoxins are human carcinogens, the most potent genotoxic agent being Aflatoxin B1 (AFB1). AFB1primarily targets the liver, where it is processed by the enzymes CYP1A2 and CYP3A4. These enzymes lead to DNA malformation and numerous mutations, particularly in the p53 gene, which functions as a tumor suppressor [5].

AFB1 is mutagenic and causes chromosomal aberrations and malformations such as sister‒chromatid alternations, DNA synthesis, micronuclei, and breakage of chromosomal strands, aneuploidy, stickiness, and ring chromosomes [6]. In addition, mutations can occur in DNA, RNA, and proteins. Both cytological research and molecular approaches have been utilized to determine whether AFB1 has a genotoxic effect on wheat plants. Different kinds of chromosomal abnormalities, including chromosome stickiness, outside bivalents, bridges, laggards, unequal division, and micronuclei, have been observed during meiosis [7].

Gel electrophoresis of proteins has become a standard method, is widely recognized, and possesses significant capabilities for use across various biological fields. SDS‒PAGE is a commonly employed technique for the qualitative analysis of protein mixtures. The application of this technique is highly advantageous for monitoring, isolating and purifying proteins. The procedure relies on the segregation of proteins depending on their mass, hence facilitating the estimation of the relative molecular mass of proteins [8]. DNA markers such as RAPD and ISSR markers have proven to be valuable tools for quickly assessing genetic variation. This is especially beneficial in situations where genome sequence information is unavailable [9].

In recent years, the fields of flow and image cytometry have gradually advanced, leading to the development of a practical, fast, and efficient technique for quantifying the nuclear DNA content within cell nuclei [10]. One possible utilization of image cytometry is to assess the cytotoxicity of environmental risks to biological systems. A significant source of environmental hazard is mycotoxins, which are toxic secondary products of certain fungi found in agricultural commodities. These toxins can cause severe toxicity in animals and humans, leading to a significant decrease in crop productivity [11].

## Results

### Cytological studies

#### Cytological effects of different concentrations of AFB1 on the mitotic cell division of *Vicia faba* root tips after exposure for 3 h

**Fig. 1:**
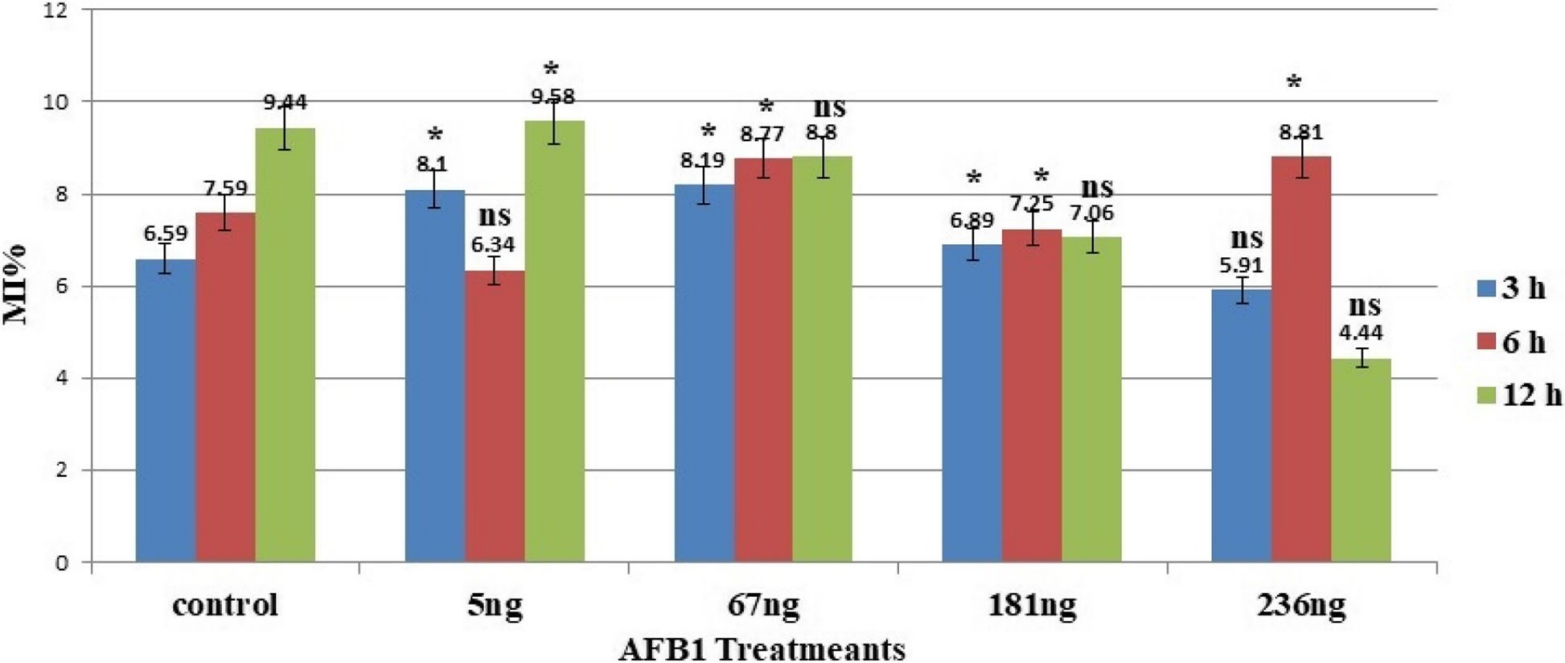
Ratio of mitotic index after treatment of *Vicia faba* root tips with three concentrations of AFB1; *: significant, ns: not significant

The root tips of *Vicia faba* plants were exposed to four different concentrations (5 ng/ml, 67 ng/ml, 181 ng/ml, and 236 ng/ml) of AFB1 for 3 h. The mitotic indices of plants treated with different concentrations of AFB1 significantly increased. However, the mitotic indices of the plants treated with 236 ng of AFB1 did not decrease significantly compared with those of the control plants (Fig. 1).

**Fig. 2:**
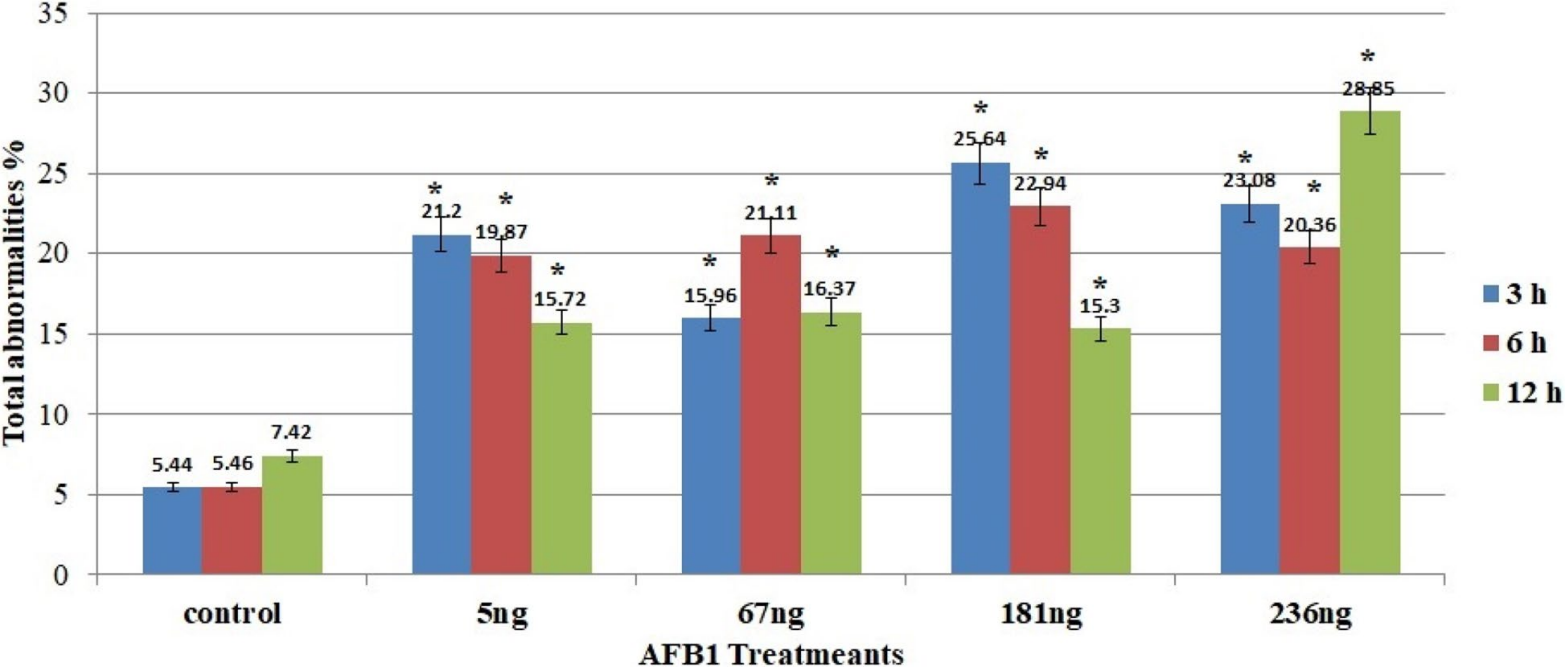
Percentage of total aberrations after *Vicia faba* root tips treatments with three concentrations of AFB1; *: significant, ns: not significant

The percentage of the phase index in each mitotic phase either increased or decreased with increasing concentrations of AFB1. Compared with that in the control group, the frequency of this phase decreased as the aflatoxin concentration increased during prophase. In contrast, at metaphase and anaphase, the frequency of both phases increased compared with that of the control. Additionally, the telophase index percentage either increased or decreased in the AFB1 treatment group. Table 1 and Fig. 2 display the percentages of abnormalities for all concentrations of AFB1 in all mitotic phases for *Vicia faba* root tips.

**Table 1:** Mitotic index, normal and abnormal phase indices, total abnormalities in non-dividing and dividing cells after treating *Vicia faba* root tips with four concentrations of AFB1, Et = Exposure time (hours)

Treatment		%MI	Phase index								% Total abnormal	
			**% prophase**		**% Metaphase**		**% Anaphase**		**% Telophase**		**Interphase**	**Mitosis**
**Concn**	**ET**		**mitotic**	**Abn**	**mitotic**	**Abn**	**Mitotic**	**Abn**	**mitotic**	**Abn**		
**Control**	**3 h**	**6.59 ± 0.32**	**44.97**	**0.00**	**23.51**	**2.25**	**10.41**	**0.54**	**20.52**	**2.65**	**0.00**	**5.44 ± 1.60**
	**6 h**	**7.59 ± 0.46**	**47.66**	**0.00**	**28.75**	**2.14**	**8.36**	**1.52**	**15.20**	**1.80**	**0.00**	**5.46 ± 1.44**
	**12 h**	**9.44 ± 0.40**	**50**	**0.00**	**25.80**	**3.55**	**11.44**	**1.73**	**12.76**	**2.14**	**0.04 ± 0.03**	**7.42 ± 0.86**
**5 ng**	**3 h**	8.10 ± 0.52*	**34.29**	**0.00**	**28.26**	**10.07**	**13.48**	**3.43**	**23.97**	**7.70**	0.30 ± 0.08*	21.20 ± 1.88*
	**6 h**	**6.34 ± 0.34ns**	**36.69**	**0.47**	**23.39**	**6.99**	**25.00**	**6.65**	**14.92**	**5.76**	0.60 ± 0.09*	19.87 ± 2.25*
	**12 h**	9.58 ± 0.43 *	**26.48**	**0.64**	**35.08**	**7.99**	**17.53**	**3.21**	**20.88**	**3.88**	0.28 ± 0.09*	15.72 ± 1.50*
**67 ng**	**3 h**	8.19 ± 0.26*	**39.46**	**0.00**	**28.69**	**7.01**	**14.49**	**5.26**	**17.36**	**3.69**	0.12 ± 0.04*	15.96 ± 1.27*
	**6 h**	8.77 ± 0.37*	**30.77**	**0.14**	**32.10**	**9.74**	**9.80**	**7.18**	**27.21**	**4.05**	0.18 ± 0.06*	21.11 ± 3.14*
	**12 h**	**8.80 ± 0.37ns**	**31.51**	**0.76**	**26.54**	**6.68**	**17.48**	**3.02**	**24.47**	**5.91**	0.24 ± 0.06*	16.37 ± 2.03*
**181 ng**	**3 h**	6.89 ± 0.30*	**37.30**	**0.00**	**24.24**	**8.05**	**17.45**	**7.27**	**21.01**	**10.32**	0.20 ± 0.09*	25.64 ± 2.08*
	**6 h**	7.25 ± 0.28*	**35.21**	**0.00**	**33.07**	**11.41**	**11.00**	**4.99**	**19.76**	**6.54**	0.10 ± 0.04*	22.94 ± 2.12*
	**12 h**	**7.06 ± 0.31ns**	**37.05**	**0.00**	**26.80**	**7.13**	**14.66**	**3.45**	**21.49**	**4.72**	0.22 ± 0.05*	15.3 ± 1.21*
**236 ng**	**3 h**	**5.91 ± 0.28 ns**	**29.63**	**0.28**	**26.44**	**11.09**	**14.65**	**5.07**	**28.75**	**6.64**	0.24 ± 0.06*	23.08 ± 2.02*
	**6 h**	8.81 ± 0.37*	**36.32**	**0.00**	**28.23**	**7.90**	**13.45**	**3.92**	**22.00**	**8.54**	**0.00**	20.36 ± 1.52*
	**12 h**	**4.44 ± 0.27ns**	**35.80**	**0.25**	**24.04**	**9.10**	**16.84**	**9.84**	**23.15**	**9.66**	0.31 ± 0.07*	28.85 ± 3.15*

Total number of observed cells = 2000, ns = not significant at 0.05 level from control, * = the two means are significantly different at the 0.05 level

**Fig. 3:**
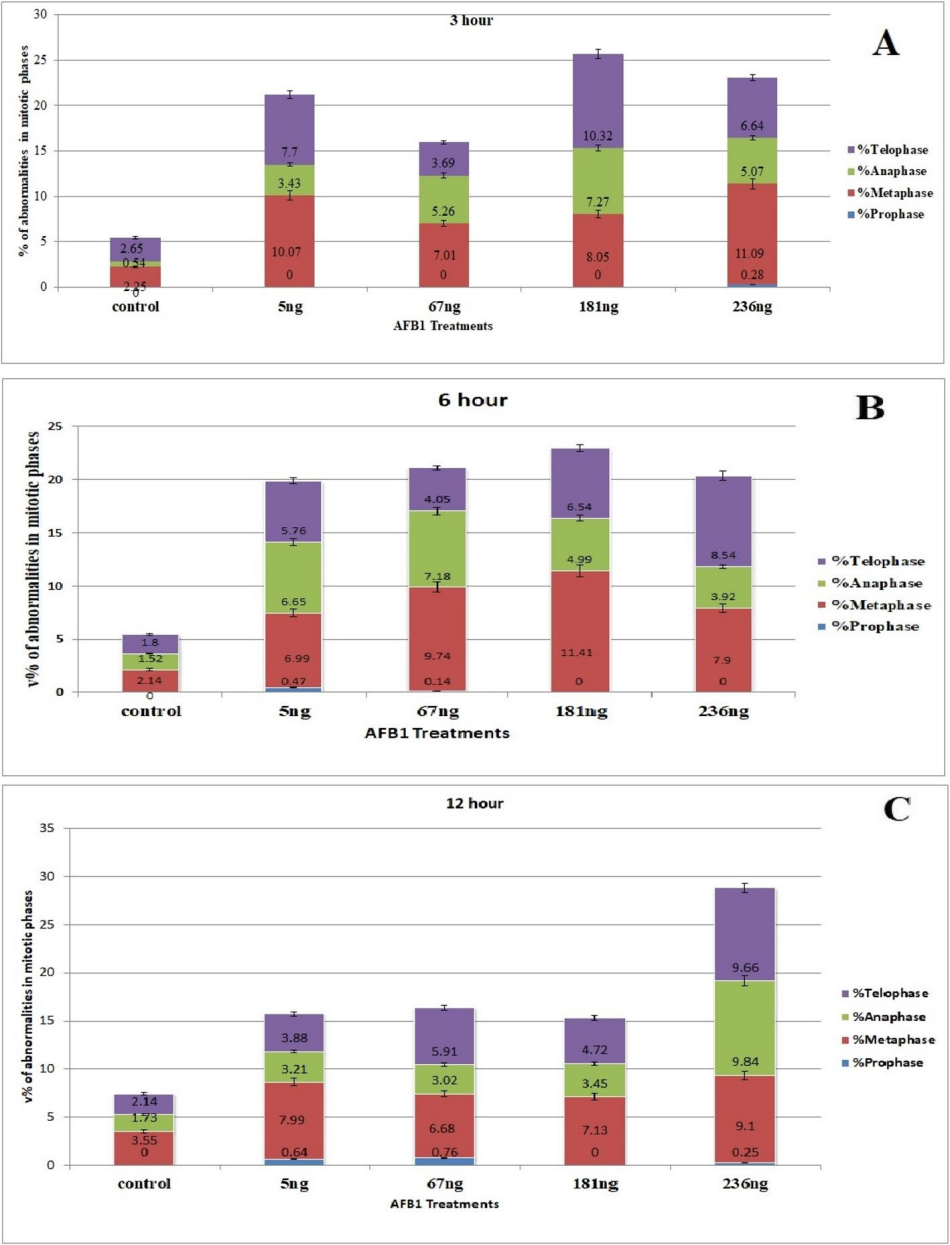
**A-C** Ratio of aberrations in mitotic phases showing abnormalities after *Vicia faba* root tips treatments for 3h, 6h and 12 h with different AFB1 concentrations

**Fig. 4:**
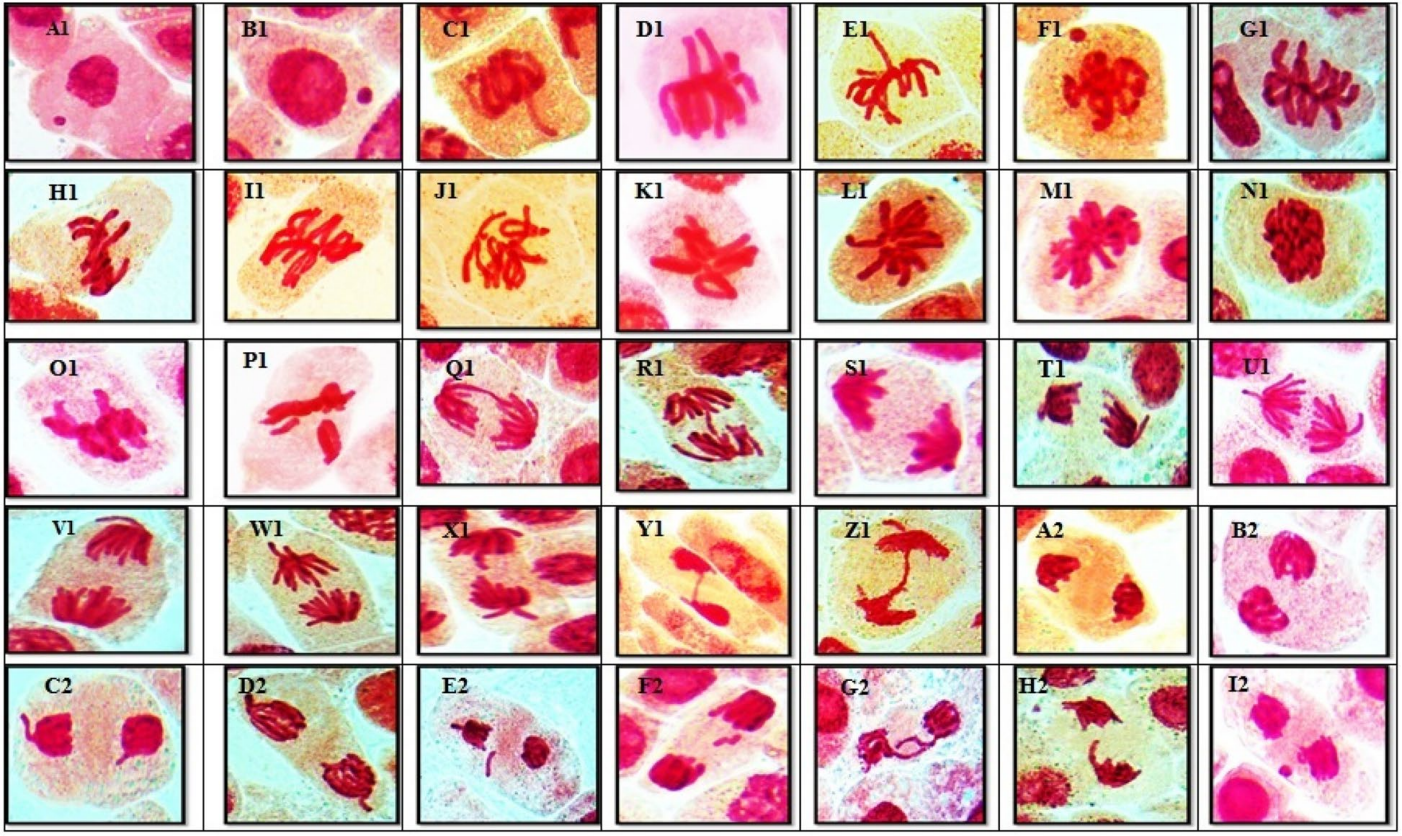
(A1 – I2): Kinds of mitotic abnormalities excited by AFB1 treated the root tips cells of *Vicia faba* with (5, 67, 181 and 236ng\ml −3h). (A1 &B1) micronucleus at interphase. (C1_E1)disturbed at metaphase. (F1) micronucleus at metaphase. (G1&I1) oblique at metaphase. (J1&K1) ring at metaphase. (L1&M1) star at metaphase. (N1) stickiness at metaphase.(O1&P1)two groups at metaphase. (Q1&R1) bridge at anaphase.(S1& T1) diagonal at anaphase.(U1&V1) disturbed at anaphase. (W1&X1) late seperation at anaphase.(Y1&Z1) bridge at telophase. (A2&B2) diagonal at telophase. (C2 &D) disturbed at telophase. (E2&F2) laggard at telophase.(G2&H2) late seperation at telophase. (I2) micronucleus at telophase (X = 1000)

For each concentration of AFB1, different chromosomal aberrations, as well as aberrations at interphase, were observed in each mitotic phase these aberrations are illustrated on Figs. 3A and 4. The results indicated that various abnormalities occurred in both non-dividing cells during interphase and dividing cells during the mitotic phasewhen different concentrations of AFB1 were applied to *Vicia faba* root tips. During interphase, variations in micronucleus size were observed, with both tiny and large micronuclei present. The percentage of abnormalities increased with increasing concentrations of AFB1. Many abnormalities, such as stickiness, two groups, ring, star-metaphase, disturbance, micronucleus, and oblique abnormalities, are detected during metaphase. In anaphase, chromosome aberrations, including late separation, diagonal, bridge, laggard, disturbed, and micronucleus, were observed on Fig. 4. Additionally, various chromosome aberrations, such as bridge laggard, diagonal, late separation, and disturbance, observed in telophase are illustrated on Fig. 4.

#### Cytological effects of different concentrations of AFB1 on the mitotic cell segregation of *Vicia faba* root tips after exposure for 6 h

The root tips of *Vicia faba* were exposed to four concentrations of AFB1for 6 h. Compared with those of the normal group, the mitotic indices of the groups treated with different concentrations of AFB1 were measured. However, the group treated with 5 ng of AFB1 did not significantly decrease, as indicated by a t test at a significance level of 0.05, as shown in Fig. 1. The percentage of the phase index in each mitotic phase either increased or decreased with increasing concentrations of AFB1. Compared with that in the control group, AFB1 frequency decreased with increasing AFB1 concentration at prophase. Compared with those of the control cells, the frequencies of metaphase and telophase cells presented varying phase indices. The percentage of the anaphase index increased with all concentrations of AFB1.

**Fig. 5:**
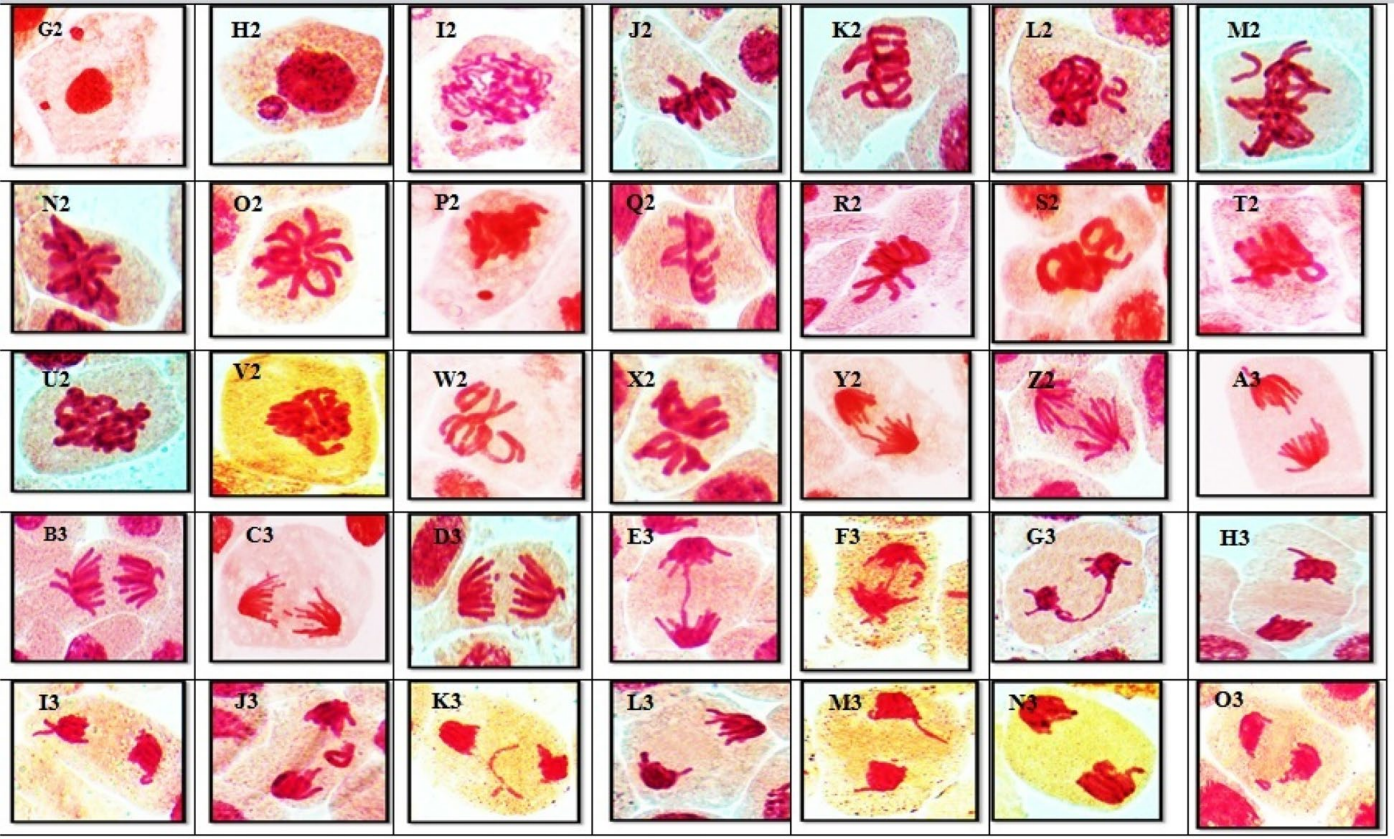
(G2 – O3): Kinds of mitotic abnormalities excited by AFB1 treated the root tips cells of *Vicia faba* with (5, 67, 181 and 236ng\ml −6h). (G2 &H2) micronucleus at interphase. (I2) micronucleus at Prophase. (J2&K2) disturbed at metaphase. (L2&M2) non congression at metaphase. (N2&O2) star at metaphase. (P2) micronucleus at metaphase. (Q2&R2) oblique at metaphase. (S2&T2) ring at metaphase. (U2&V2) stickiness at metaphase.(W2&X2)two groups at metaphase. (Y2&Z2) bridge at anaphase.(A3&B3) disturbed at anaphase. (C3) laggard at anaphase.(U1&V1) Micronucleus at anaphase.(E3-G3) bridge at telophase. (H3 &I3) disturbed at telophase. (J3&K3) laggard at telophase. (L3&M3) late seperation at telophase (N3) diagonal at telophase. (o3) Ring at telophase (X = 1000)

The percentages of abnormalities for all concentrations of AFB1 in all mitotic phases for *Vicia faba* root tips are presented in Table 1 and Fig. 2. The ratio of total aberrations increased with all concentrations of AFB1. Different chromosomal aberrations were observed for each concentration of aflatoxin in each mitotic phase, as were aberrations at interphase, as illustrated on Figs. 3B and 5. The abnormalities observed at interphase were micronuclei of varying sizes (small and/or large). At prophase, the frequency of micronucleus aberrations decreased with increasing concentrations of AFB1. Many abnormalities, including stickiness, two groups (ring, star-metaphase), disturbances, micronuclei, oblique, and non-congression, are detected during metaphase. These abnormalities were observed on Fig. 5. Additionally, various chromosome aberrations, such as late separation, diagonal, bridge, laggard, disturbed, and micronucleus aberrations, are observed at the anaphase stage. At telophase, numerous chromosome aberrations, including disturbances, bridge, diagonal, laggard, and late separations, were observed, all of which are depicted on Fig. 5.

#### Cytological effects of different concentrations of AFB1 on mitotic cell segregation in the root tips* of Vicia faba* after 12 h of exposure

The root tips of *Vicia faba* were treated with four different concentrations of AFB1 for 12 h. According to the t test, with a significance level of 0.05, the mitotic indices of the various AFB1 treatment groups were not significantly lower, except for those of the 5 ng treatment group, which were significantly greater than those of the control group, as shown in Fig. 1. The percentage of the phase index in each mitotic phase increased or decreased with increasing concentrations of AFB1. Compared with that in the control, the frequency of prophase decreased in response to all AFB1 treatments. While the frequency of metaphase tends to vary, the ratio of anaphase to telophase indices increased with all concentrations of AFB1.

**Fig. 6:**
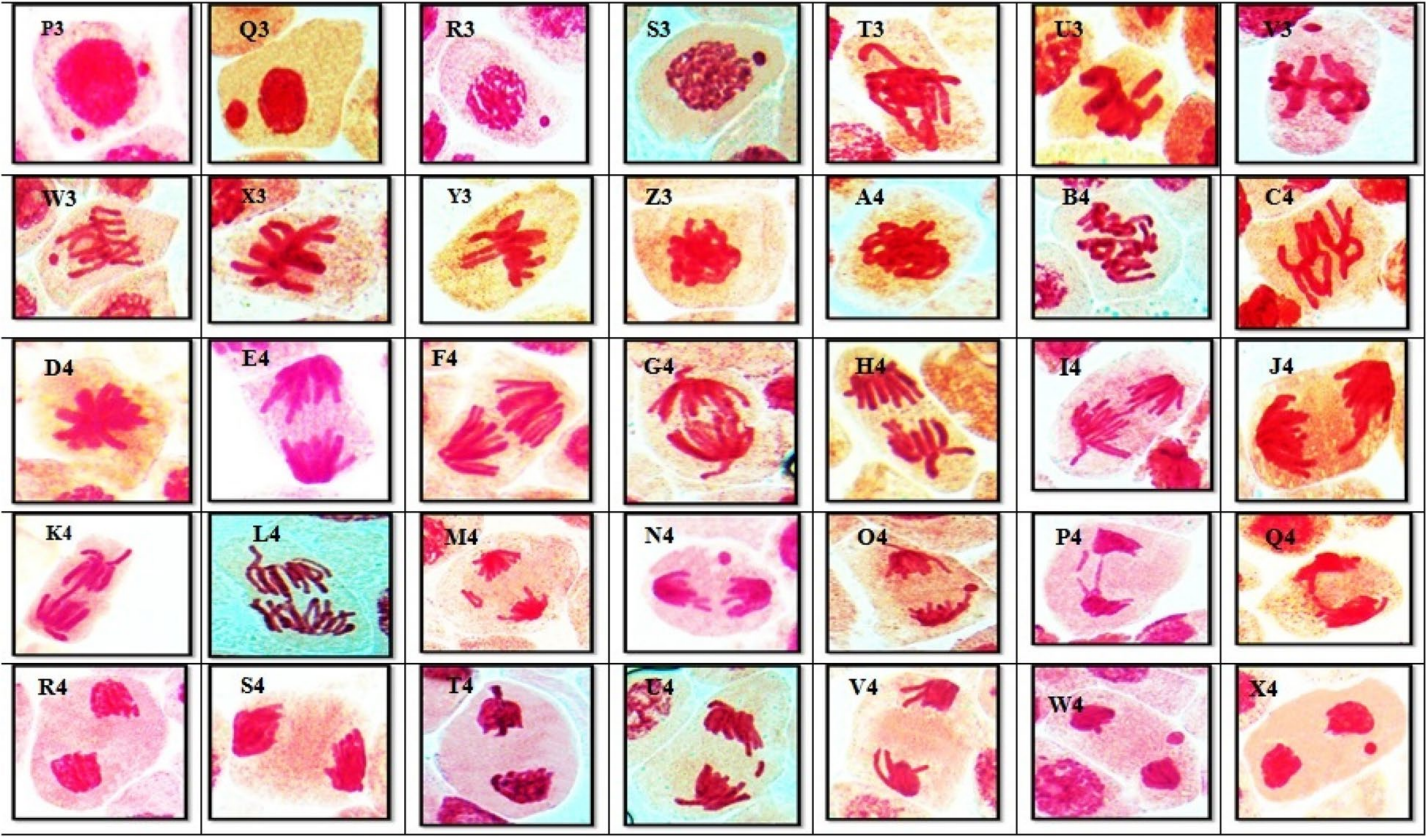
(P3 – X4): Kinds of mitotic abnormalities excited by AFB1 treated the root tips cells of *Vicia faba* with (5, 67, 181 and 236ng\ml −12h). (P3 &Q3) micronucleus at interphase. (R3&S3) micronucleus at Prophase. (T3&U3) disturbed at metaphase. (V3&W3) micronucleus at metaphase. (X3&Y3) oblique at metaphase. (Z3&A4) stickiness at metaphase. (B4&C4) two groups at metaphase. (D4) star at metaphase. (E4&F4) digonal at anaphase.(G4&H4) disturbed at anaphase. (I4&J4) late seperation at anaphase.(K4&L4) Bridge at anaphase. (M4) laggard at anaphase. (N4&O4) Micronucleus at anaphase. (P4&Q4) bridge at telophase. (R4 &S4) diagonal at telophase. (T4) disturbed at telophase. (U4) laggard at telophase. (V4) late seperation at telophase (W4&X4) Micronucleus at telophase (X = 1000)

The percentages of abnormalities for all concentrations of AFB1 in all mitotic phases for *Vicia faba* root tips are shown in Table 1 and Fig. 2. The ratio of total aberrations increased at all concentrations of AFB1. Different chromosomal aberrations were observed in each mitotic phase in addition to aberrations at interphase, as illustrated on Figs. 3C and 6. Abnormalities observed at interphase included micronuclei of varying sizes (small and/or large). During metaphase,various aberrations, such as stickiness, ring formation, star metaphase, disturbance, and micronucleidetected. Additionally, at the anaphase stage, chromosome aberrations diagonal, bridge, laggard, disturbance, and micronuclei were observed on Fig. 6. Various chromosome aberrations, such as bridge formation, laggard chromosomes, diagonal patterns, late separation, disturbance, and micronuclei, were observed at telophase, as illustrated on Fig. 6.

### Seed protein electrophoresis

**Fig. 7:**
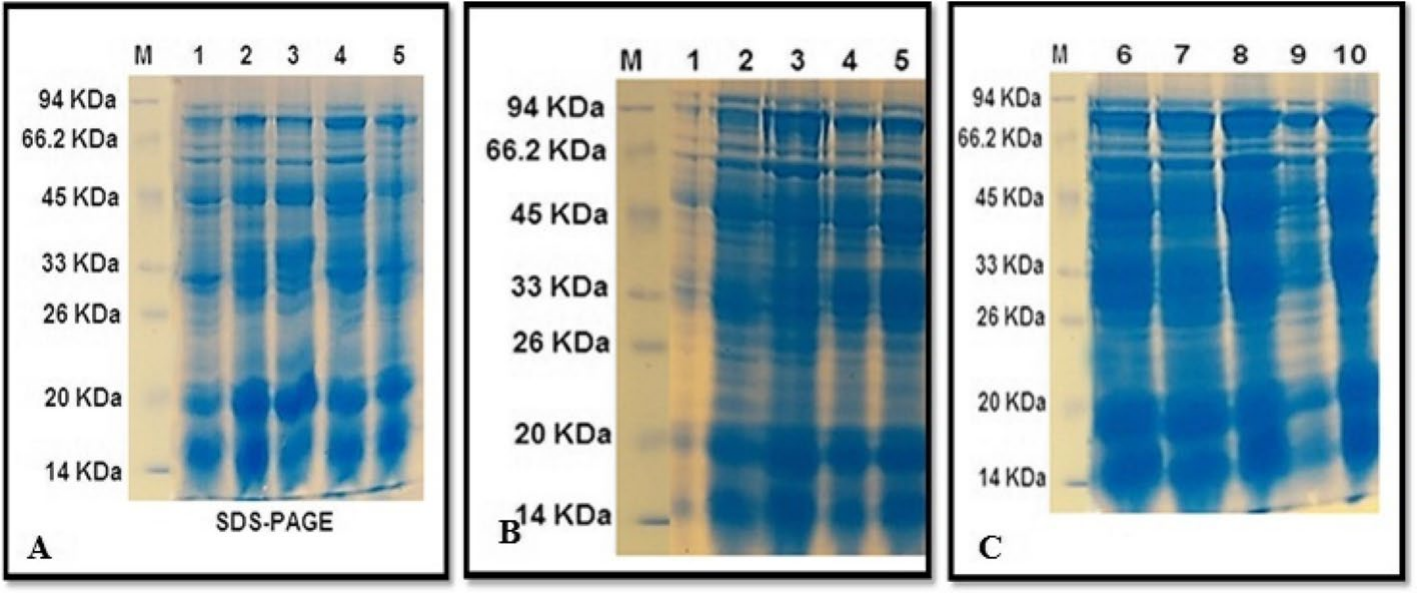
Electrophotograph of SDS-PAGE of total proteins of M1 seeds of *Vicia faba* treated with different concentrations of AFB1. M = Molecular weight. **A** 1= Control. 2= 3h 5 ng. 3= 3h 67 ng. 4= 3h 181 ng. 5= 3h 236 ng. **B** 1= Control. 2= 6h 5 ng. 3= 6h 67 ng. 4= 6h 181 ng. 5= 6h 236 ng. **C** 6= Control. 7= 12h 5 ng. 8= 12h 67 ng. 9= 12h 181 ng. 10= 12h 236 ng

The electrophoretic analysis of seed protein extracts from the thirteen samples treated with different concentrations of AFB1 (67 ng, 181 ng, 236 ng, and 5 ng) as positive controls, as well as water as a negative control after 3, 6, and 12 h, is shown in Fig. 7. Each sample exhibited a distinct electrophoretic pattern, with both qualitative and quantitative changes. These changes were evident through the emergence of new bands and the disappearance of existing bands. In total, 14 bands were detected, comprising nine monomorphic bands, two unique bands, and three non-unique bands.

For an exposure time of 3 h, the 14 detected bands had molecular weights ranging between 14 and 94 kDa. These bands are not always expressed in all the samples. The protein profile revealed ten monomorphic bandsthat were common to all the different seeds subjected to different AFB1 treatments. However, there were two bands specific to a particular treatment, the 26 kDa band disappeared only in seeds treated with 67 ng as a negative molecular marker. dditionally, the 38 kDa band disappeared in seeds treated with water, which was used as a negative control. These bands could serve as valuable positive molecular indicators for seeds treated with AFB1.

After 6 h of exposure, the protein profile revealed eleven monomorphic bands. Two bands were specific to a particular treatment; bands 38 and 43 kDa were present only in seeds treated with water as a negative control. These bands could be used as unique molecular indicators for AFB1 -treated seeds.

Additionally, after 12 h of exposure, nine monomorphic bands, two unique bands, and three non-unique bands were observed, two of which (26 and 38 kDa) were specific to a particular treatment. Furthermore, a band with a molecular weight of 27 kDa was present only in seeds treated with water as a negative control. Interestingly, the band with a molecular weight of 38 kDa disappeared in seeds exposed to water as a negative controlThese bands could serve as valuable molecular markers.

In the 3-h treatment, the highest percentage of similarity reached 100%, which was achieved with seeds exposed to AFB1 at a concentration of 181 ng. Consistent with the results for the seeds treated for 3 h, those subjected to 5 ng of AFB1 presented fourteen similar bands and no dissimilar bands. Furthermore, in the 6-h treatment, the percentage with the highest similarity was also 100%, which was observed in both the control seeds and those treated with AFB1 (5 ng, 67 ng, and 181 ng). These seeds presented fourteen similar bands and no dissimilar bands. In the case of the 12-h treatment, the highest percentage of similarity was recorded with seeds treated with 236 ng of AFB1. Similarly, the seeds treated with 181 ng of AFB1 during the 12-h exposure presented thirteen similar bands andno dissimilar bands.

The highest percentage was 36.36%, detected in four samples: two after exposure for 3 h (5 ng and 181 ng) and two after exposure for 12 h (181 ng and 236 ng). Additionally, the GTS percentage data were consistent with the presence of developing bands and the absence of original bands compared with those of the control. For the 6-h exposure time, the maximum percentage of GTS (81.81%) was found in the root tips of *Vicia faba* exposed to the highest concentration of aflatoxin (236 ng); furthermore, the highest percentage of genomic stability for the 6-h exposure time was shown in the treatment with 236 ng of AFB1 (81.81%). Moreover, *Vicia faba* treated with 5 ng, 181 ng, or 236 ng of AFB1 had a GTS percentage of 63.63%.

### Molecular analysis

#### Random Amplified Polymorphic DNA (RAPD) analysis

**Fig. 8:**
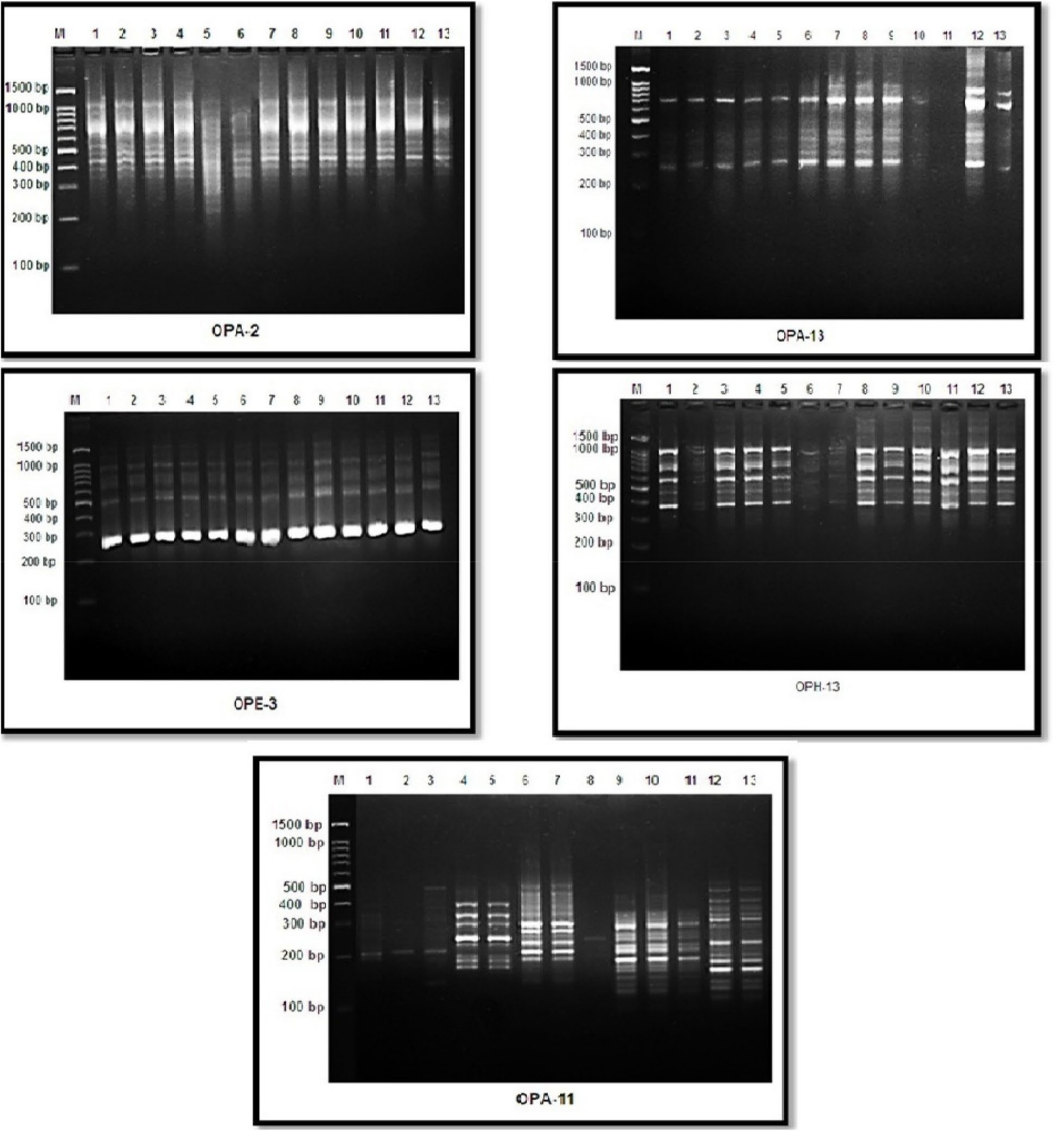
The amplified DNA fragments that produced by five primers RAPD for the thirteen samples treated with different concentrations of AFB1. M = Molecular size. 1= Control. 2= 3h 5 ng. 3= 3h 67 ng. 4= 3h 181 ng. 5= 3h 236 ng. 6= 6h 5 ng. 7= 6h 67 ng. 8= 6h 181 ng. 9= 6h 236 ng. 10= 12h 5 ng. 11= 12h 67 ng. 12= 12h 181 ng. 13= 12h 236 ng

Five primers were used in this study to differentiate between the young green leaves of growing *Vicia faba* seeds that were exposed to various exposure times and doses of AFB1. As depicted in Fig. 8, there were a total of 51 well-defined and successful bands.

Among the 51 bands, twelve were common to all the samples, either the control or treated samples, and 28 were non-unique polymorphic bands, whereas eleven were unique polymorphic bands. One polymorphic band appeared with the OPA-2 primer, seven polymorphic bands with the OPA-13 primer, one polymorphic band presented with the OPE-3 primer, four polymorphic bands with the OPH-13 primer, and twenty-six polymorphic bands with the OPA-11 primer. The percentage of polymorphismsfor each primer is shown in Table 2.

**Table 2:** Count and kinds of the amplified DNA bands and the percentage of the total polymorphism generated by five primers for samples treated with different concentration of AFB1

Primer code	Molecular weight range	Monomorphic bands	Polymorphic bands		Total bands	Polymorphism%
			**unique**	**Non unique**		
**OPA-2**	393-1023bp	5	0	1	6	16.6%
**OPA-13**	255-909bp	5	2	5	7	100%
**OPE-3**	290-1361bp	5	0	1	5	100%
**OPH-13**	332-1012bp	3	1	3	7	57.1%
**OPA-11**	124-529bp	0	8	18	26	100%
**Average**		**2.4**	**2.2**	**5.6**	**10.2**	**58.74%**

The amplification profiles of the samples were determined via five primers: OPA-2, OPA-13, OPE-3, OPH-13, and OPA-11. RAPD markers were obtained by fingerprinting thirteen control samples and samples treated for 3 h, 6 h, or 12 h with different concentrations of AFB1 (5, 67, 181, or 236 ng). The molecular weights of these bands ranged from 124 to 1361 bp. Additionally, the average percentage of polymorphic bands in the investigated treatments was 58.74%. Concerning the percentage of polymorphisms in the samples treated with different exposure times and AFB1 concentrations, the maximum percentage was 100% for both primers, OPA-11 and OPA-13, with sequences of 5´ CAATCGCCGT 3` and 5´ CAGCACCCAC 3`.

The GTS percentages were determined on the basis of the presence of developed bands and the absence of original bands compared with those of the control (Table 3). The maximum percentages of GTS at 3 h, 6 h, and 12 h were 73.91%, 52.17%, and 60.87%, respectively.

**Table 3:** Percentages of genomic template stability (relating to RAPD profiles) for treated *Vicia faba* samples with various concentrations of AFB1 and at three exposure times; a: refers to presence of new bands, b: lacking of normal bands and a + b: number of polymorphic bands

Marker	Primer name	Control (Egypt)	3h								6h								12h							
			**5 ng**		**67 ng**		**181 ng**		**236 ng**		**5 ng**		**67 ng**		**181 ng**		**236 ng**		**5 ng**		**67 ng**		**181 ng**		**236 ng**	
			a	b	a	b	a	b	a	b	a	b	a	b	a	b	a	b	a	b	a	b	a	b	a	b
**RAPD**	OP-A2	6	0	0	0	0	0	0	0	1	0	1	0	0	0	0	0	0	0	0	0	0	0	0	0	0
	OP-A13	2	0	0	0	0	0	0	2	0	3	0	4	0	4	0	4	0	0	0	0	2	4	0	1	0
	OP-E3	4	1	0	1	0	1	0	0	0	0	0	0	0	1	0	1	0	0	0	0	0	0	0	0	0
	OP-H13	6	0	1	0	0	0	0	0	0	0	2	0	3	0	0	0	0	0	0	1	0	0	0	0	0
	OP-A11	5	0	4	2	4	5	4	6	4	6	3	6	3	1	5	6	3	6	3	4	3	9	3	9	3
	Total number of bands	23	1	5	3	4	7	5	8	5	9	6	10	6	6	5	11	3	6	3	5	5	13	3	10	3
	a + b	––-	6		7		12		13		15		16		11		14		9		10		16		13	
	GTS %	100%	73.91%		69.56%		56.52%		43.47%		34.78%		30.43%		52.17%		39.13%		60.86%		56.52%		30.43%		43.47%	

#### Inter Simple Sequence Repeat (ISSR) DNA analysis

**Fig. 9:**
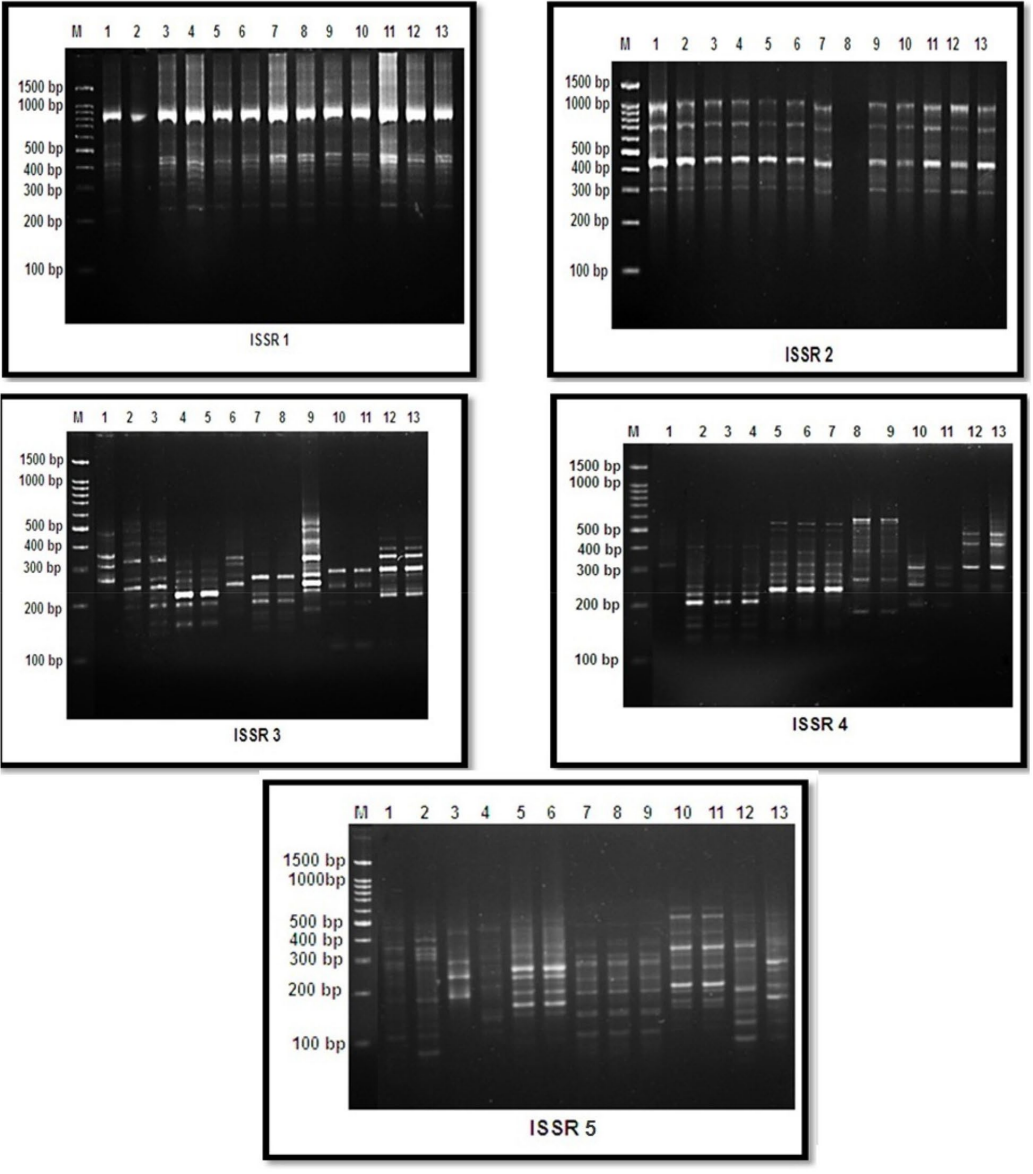
The amplified DNA fragments that produced by five primers of ISSR for the thirteen samples treated with different concentrations of AFB1. M = Molecular size . 1= Control. 2= 3h 5 ng. 3= 3h 67 ng. 4= 3h 181 ng. 5= 3h 236 ng. 6= 6h 5 ng. 7= 6h 67 ng. 8= 6h 181 ng. 9= 6h 236 ng. 10= 12h 5 ng. 11= 12h 67 ng. 12= 12h 181 ng. 13= 12h 236 ng

The thirteen genomic DNA samples, which had different exposure times and various AFB1 concentrations, were amplified via five ISSR primers to differentiate between the samples. ISSR analysis was conducted with five primers: ISSR1, ISSR2, ISSR3, ISSR4, and ISSR5. These primers produce a range of DNA fragments, varying in number on the basis of the motifs included in their simple sequence repeats. The generated bands ranged from 83 to 1009 bp, with 86 detectable bands, including one monomorphic band common to all samples and 58 non-unique polymorphic bands. In total 27 unique polymorphic bands were identified, as shown in Table 4 and Fig. 9. The number of successful markers produced per primer ranged from 7 for ISSR2 to 27 for ISSR3.

**Table 4:** Count and kinds of the amplified DNA bands and the ratio of the total polymorphism produced by five primers for samples treated with different concentration of AFB1

Primer code	Molecular size range	Monomorphic bands	Polymorphic bands		Total bands	Polymorphism%
			**unique**	**Non unique**		
**ISSR1**	**195-863bp**	**1**	**4**	**5**	**10**	**90.0%**
**ISSR2**	**255-1009bp**	**0**	**6**	**1**	**7**	**100.0%**
**ISSR3**	**115-580bp**	**0**	**10**	**17**	**27**	**100.0%**
**ISSR4**	**152-630bp**	**0**	**2**	**17**	**19**	**100.0%**
**ISSR5**	**83-606bp**	**0**	**5**	**18**	**23**	**100.0%**
**Average**		**0.2**	**5.4**	**11.6**	**17.2**	**98%**

The average percentage of polymorphic bands in the samples was 98%.The maximum ratio of polymorphism percentage in the studied treatment was 100% for all primers except ISSR-1, which the minimum polymorphism percentage 90%, with the sequence 5' CTCTCTCTCTCTCTCTG 3`. Additionally, the maximum percentages of GTS at 3 h, 6 h, and 12 h were 51.5%, 24.2%, and 18.2%, respectively, as shown in Table 5.

**Table 5:** Percentages of genomic template stability (relating to ISSR profiles) for treated *Vicia faba* samples with different concentrations of AFB1 and at three exposure times; a: refers to presence of new bands, b: lacking of normal bands and a + b: number of polymorphic bands

Marker	Primer name	Control (Egypt)	3h								6h								12h							
			**5 ng**		**67 ng**		**181 ng**		**236 ng**		**5 ng**		**67 ng**		**181 ng**		**236 ng**		**5 ng**		**67 ng**		**181 ng**		**236 ng**	
			a	b	a	b	a	b	a	b	a	b	a	b	a	b	a	b	a	b	a	b	a	b	a	b
**ISSR**	ISSR 1	5	1	4	1	0	1	0	1	1	1	1	1	1	2	1	1	1	1	1	2	1	2	2	1	1
	ISSR 2	6	0	1	0	2	0	2	0	2	0	2	0	2	0	6	1	2	0	2	0	2	0	2	0	2
	ISSR 3	6	2	1	3	1	3	4	3	4	3	4	3	5	3	5	6	5	3	6	4	6	6	6	6	6
	ISSR 4	6	3	2	3	4	3	5	2	3	2	3	2	3	3	3	3	2	3	4	2	3	3	3	3	2
	ISSR 5	10	1	1	2	4	3	6	4	2	4	4	3	6	3	4	3	5	3	4	3	5	2	4	2	6
	Total number of bands	33	7	9	9	11	10	17	10	12	11	14	9	17	11	19	14	15	10	17	11	17	13	17	12	17
	a + b	––-	16		20		27		22		25		26		30		29		27		28		30		29	
	GTS %	100%	51.5%		39.4%		18.2%		33.3%		24.2%		21.2%		9.1%		12.1%		18.2%		15.2%		9.1%		12.1%	

### Flow cytometry study

#### Effect of seeds treated with different AFB1 concentrations three times on B-cell lymphoma 2 (Bcl2)

**Fig. 10:**
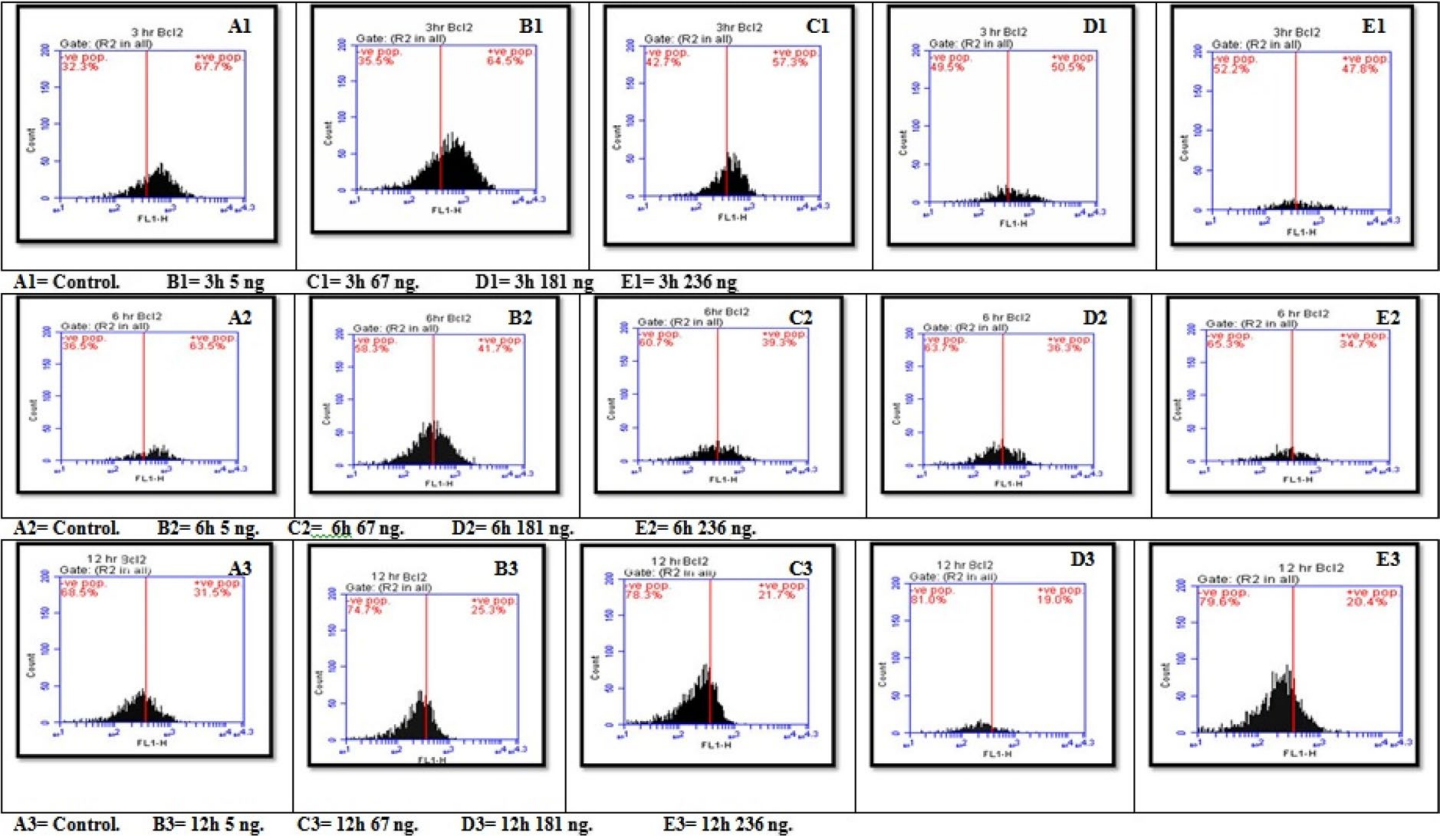
Interaction effect of seeds treated with different AFB1 concentrations and three exposure times (3, 6 and 12h) on Bcl2

The data revealed a statistically significant effect of different AFB1 concentrations and durations on bcl2 expression. All pairwise comparisons for the AFB1 groups were significant except for those involving the 6-h 181 ng group with 67 ng and 236 ng groups and the 12 h 236 ng with 67 ng and 181 ng groups. As time progressed, the bcl2 level decreased from 3 to 6 h and 12 h. The highest value was 64.50 at 3 h 5 ng, and the lowest value was 19.00 at 12 h at 181 ng. Additionally, the main effects of AFB1 concentration and time were significant. With increasing AFB1 concentration, the bcl2 level decreased. The lowest value was at 181 ng, and the highest value was 25.30 ng at 5 ng in the control (Figs. 10 and 14A).

#### Effects of three different concentrations of AFB1 on chromatin condensation in seeds

**Fig. 11:**
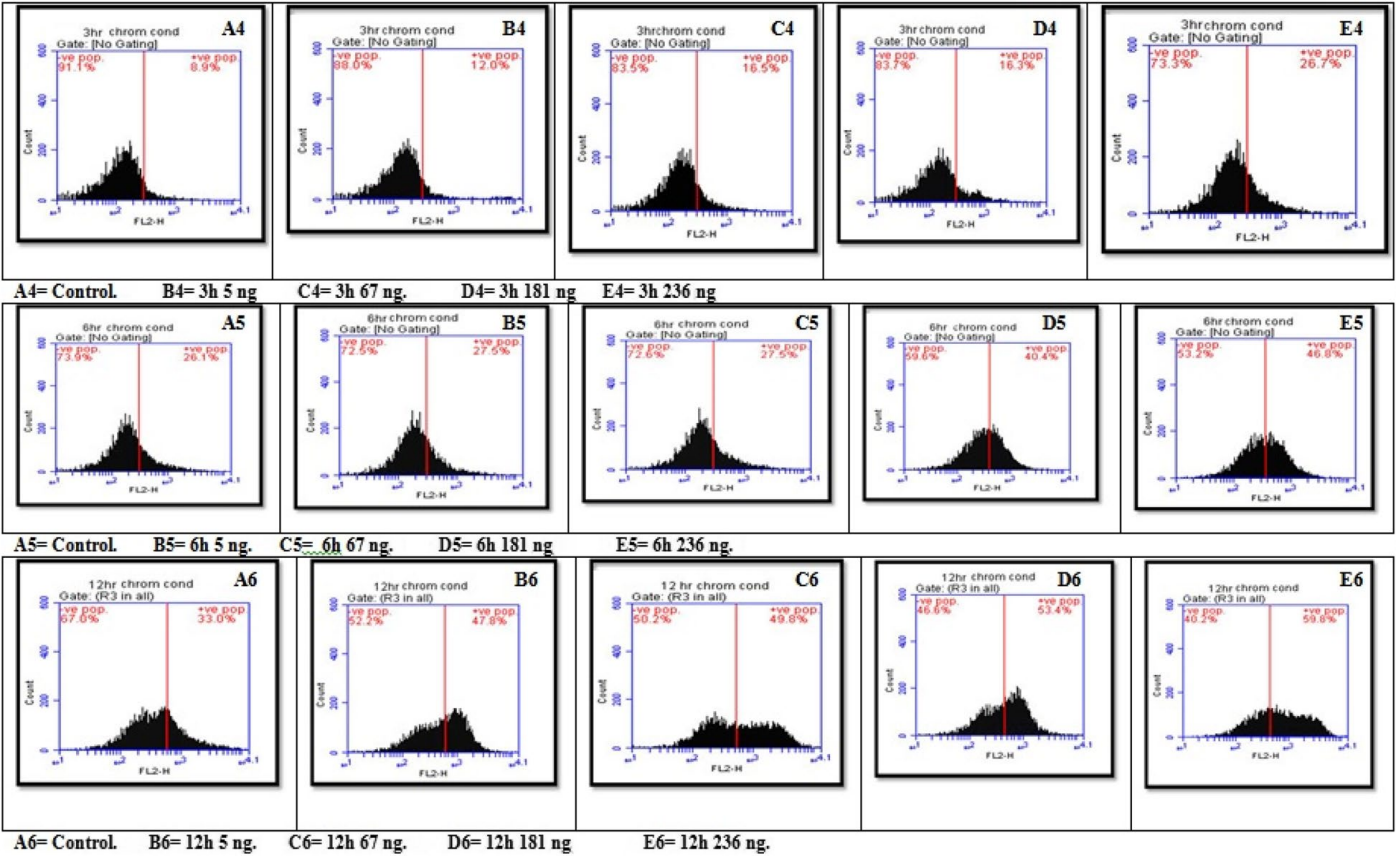
Interaction effect of seeds treated with different AFB1 concentrations and three exposure times (3, 6 and 12h) on Chromatin condensation

The results revealed a statistically significant interaction effect of different aflatoxin B1 (AFB1) concentrations and durations on chromatin condensation. All pairwise comparisons for groups of AFB1 were significant except at 3 h (67 ng with 181 ng), 6 h (control with 5 ng and 67 ng), and 12 h (5 ng with 67 ng). Since all the groups mentioned have no interaction with each other, for a simple main effect, all are significant for seeds treated with different AFB1 concentrations and times. Additionally, with increasing exposure time to AFB1, chromatin condensation levels increase, and the higher the AFB1 concentration is, the greater the degree of chromatin condensation. The highest value was 59.80 at 236 ng for 12 h. The lowest value was 12.00 at the positive control (5 ng) for a 3-h exposure time (Figs. 11 and 14B).

#### Effects of three different concentrations of AFB1 on Poly ADP‒Ribose Polymorphism (PARP) in seeds

**Fig. 12:**
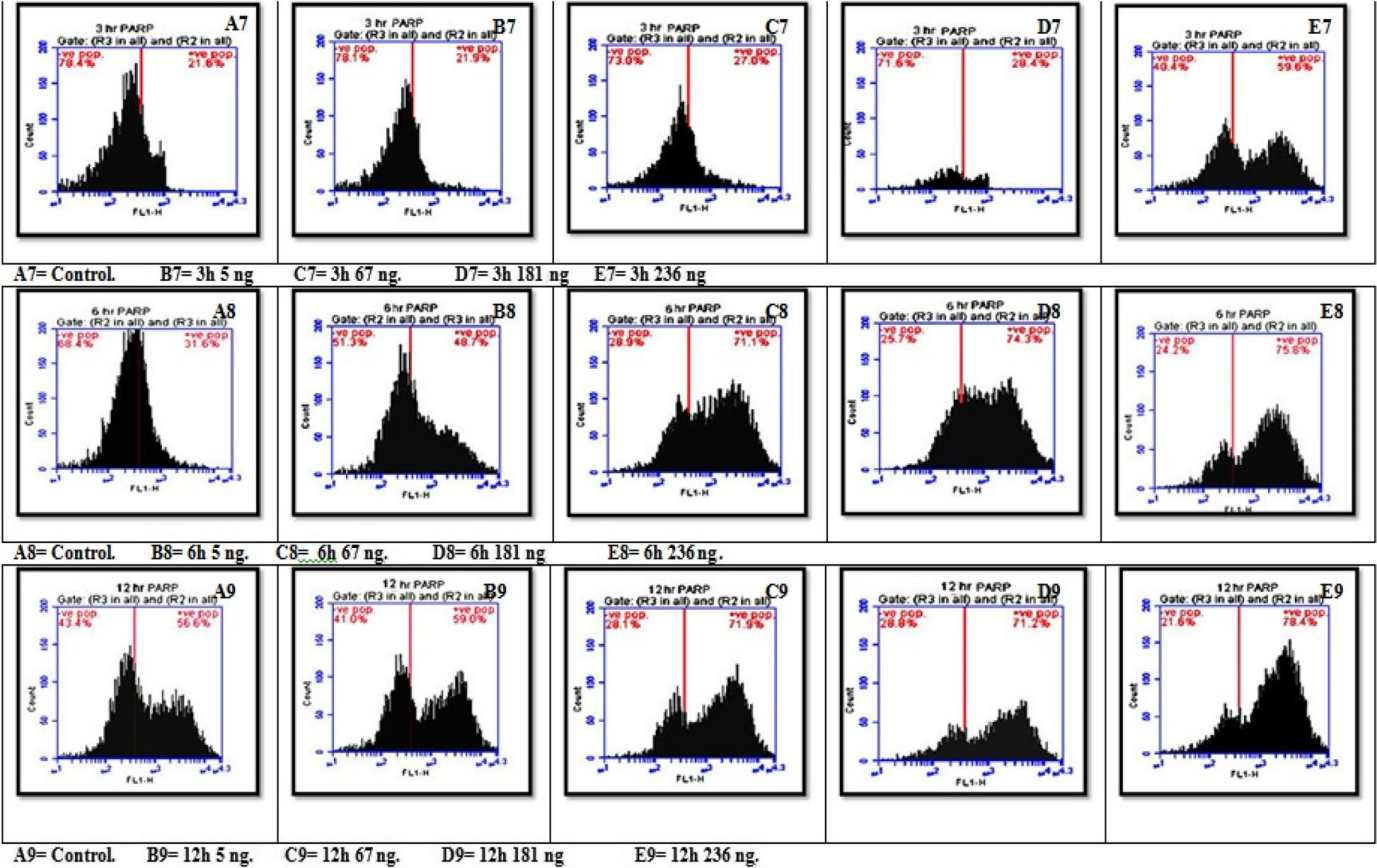
Interaction effect of seeds treated with different AFB1 concentrations and three exposure times (3, 6 and 12h) on PARP

The results revealed a statistically significant interaction effect of different aflatoxin B1 (AFB1) concentrations and exposure times on PARP. All pairwise comparisons for the AFB1 groups were significant except at 3 h (control with 5 ng and 67 ng with 181 ng), 6 h (67 ng with 181 ng and 181 ng with 236 ng), and 12 h (control with 5 ng, 67 ng with 181 ng and 236 ng and 181 ng with 236 ng). Since there was no significant effect between the previous groups all the effects were significant for seeds treated with different AFB1 concentrations and durations except between 6 and 12 h in the 67, 181, and 236 ng groups. Additionally, the PARP concentration clearly increased with increasing exposure time and AFB1 concentration,with the highest value being 78.40 at 236 ng for 12 h and the lowest at 21.90 at 5 ng and 3 h, as shown in Figs. 12 and 14C.

#### Effects of treatment of seeds with different AFB1 concentrations three times on the cell cycle

**Fig. 13:**
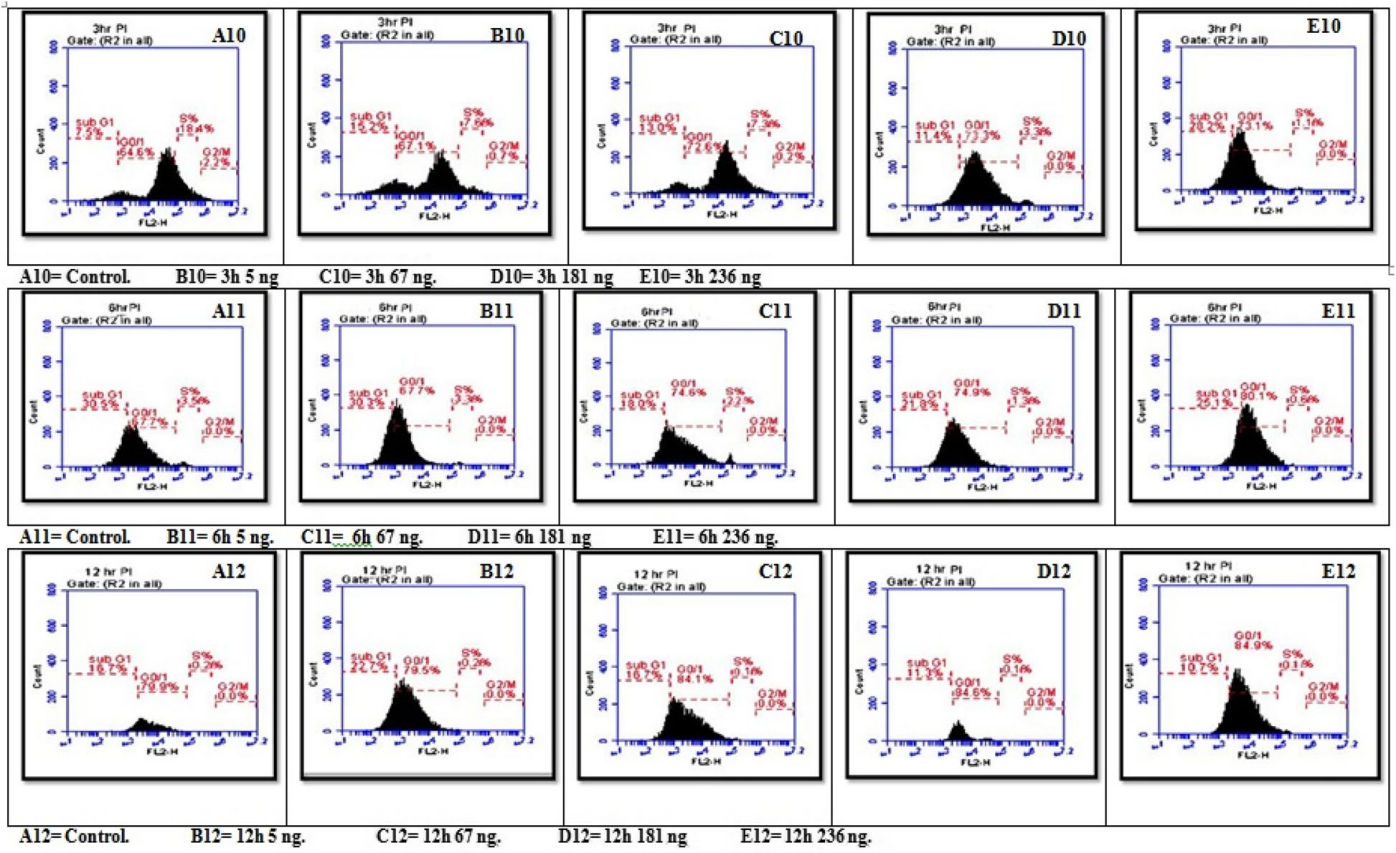
Interaction effect of seeds treated with different AFB1 concentrations and three exposure times (3, 6 and 12h) on Cell cycle

**Fig. 14:**
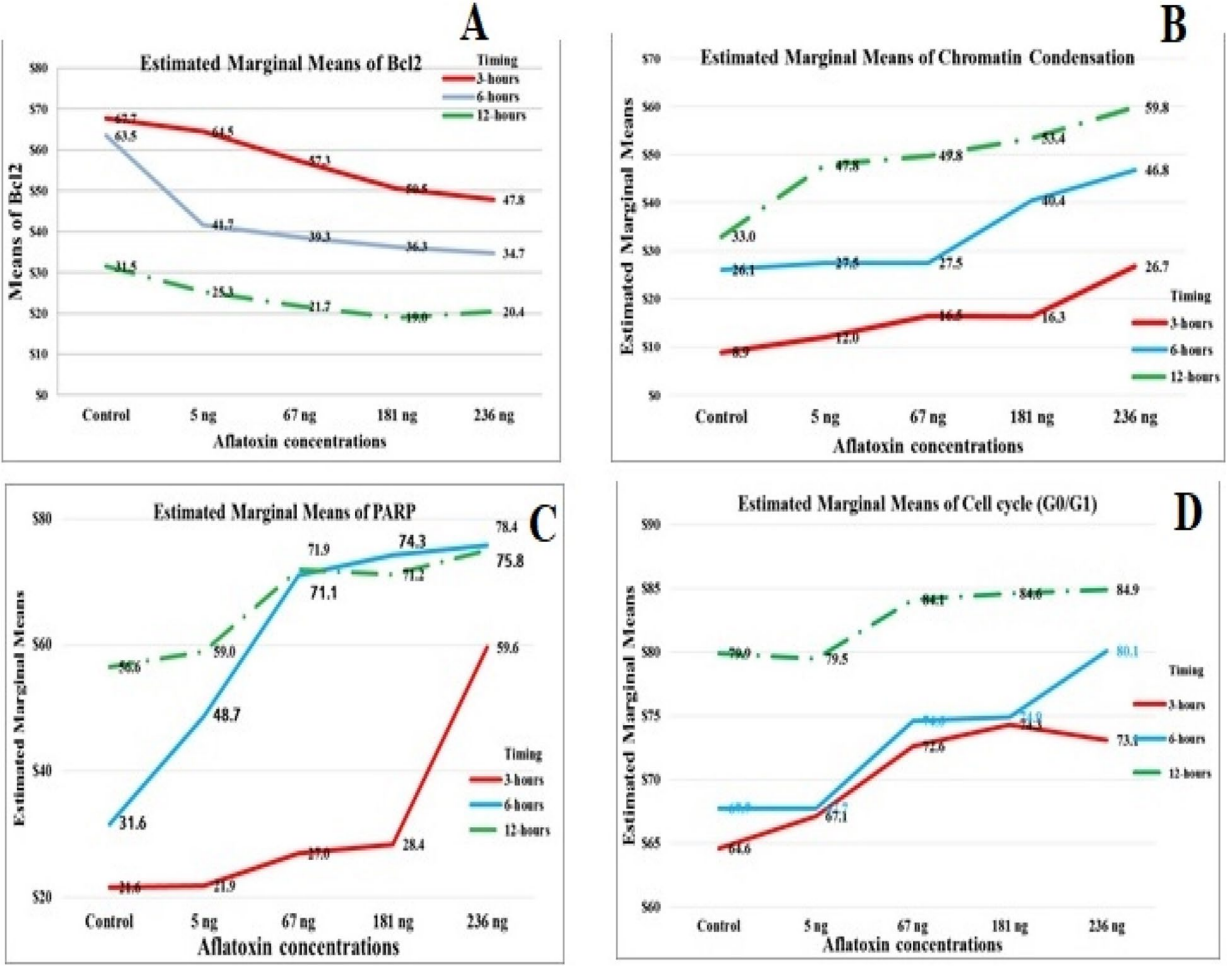
Interaction effect of seeds treated with different AFB1 concentration and three time intervals on **A**) Bcl2, **B** Chromatin condensation, **C** PARP & **D**) Cell cycle G0/1 phase

Figures 13 and 14D show a statistically significant interaction effect between different aflatoxin B1 (AFB1) concentrations and exposure times on the G0 and G1 phases. All pairwise comparisons for the AFB1 groups were significant except at 3 h for the 181 ng, 236 ng and 67 ng groups; at 6 h for the 5 ng and 181 ng control groups; and at 12 h for the 5 ng and 67 ng control groups, with 181 ng, 236 ng and 181 ng, respectively.

Compared with the results at 3 h and 6 h, the three AFB1 concentrations (5 ng, 67 ng, and 181 ng) had significant effects on the growth of seeds treated with AFB1. The impact of the studied AFB1 on the cell cycle in the G0/G1 phase revealed a progressive increase in the number of cells with higher AFB1 concentrations after longer seed treatment with B1 toxin. The number of cells increased from 64.60 in the control group to 73.10 in cells treated with 236 ng of AFB1 for the same exposure time. Additionally, the highest proportion of cells treated with 236 ng of AFB1 after 12 h was 84.90.

For the S-phase, there was a statistically significant interaction effect of different AFB1 concentrations and exposure times, except at 3 h, 5 ng, and 67 ng. Moreover, at 6 h, all the groups were not significantly different except for the control group, which received 181 ng and 236 ng, respectively. At 12 h, the groups were not significantly different from each other. Additionally, the main effects of AFB1 concentration and time were significant for all the seeds, except for the 181 ng AFB1 concentration, which differed between the 6 h and 12 h groups. The 236 ng AFB1 concentration groups were not significantly different.

Finally, the G2/M phase showed a statistically non-significant interaction effect of varying AFB1 concentrations and exposure times on the G2/M phase. All pairwise comparisons for the AFB1 groups were not significant at 3 h, except for the 181 ng and 236 ng AFB1 groups, which were significant. Furthermore, the main effects ofAFB1 concentration and time were significant for all the seeds, but in the control group, the effects in the 5 ng and 67 ng groups were not significantly different from those in the 6 h and 12 h groups. Additionally, the effects in the 181 ng and 236 ng groups were not significantly different, indicating that there was no correlation between the two groups.

Moreover, the proportions of cells involved in the DNA generation period (S_phase), G2_phase, and Mphase gradually decreased with increasing concentrations of AFB1. The number of cells in Sphase decreased from 18.40 in the normal group to 7.60, 7.30, 3.30, and 1.11 in the 5 ng, 67 ng, 181 ng, and 236 ng treatment groups, respectively, after 3 h of exposure. The number of cells exposed for 6 h decreased as the cell proportion decreased from 3.50 in the control group to 3.30, 2.20, 1.30, and 0.60 in the 5 ng, 67 ng, 181 ng, and 236 ng treatment groups, respectively. Moreover, plants exposed for 12 h had the same proportion of cells (0.20 in both the positive and negative control roots), which decreased to 0.10 for all the root cells treated with 67 ng, 181 ng, and 236 ng, respectively.

## Discussion

Aflatoxin B1 (AFB1) is widely recognized as an extremely virulent hepatocarcinogen that occurs naturally [12]. The International Agency for Research on Cancer has classified it as a Category 1A carcinogen. Cereal grains and animal feeds worldwide are susceptible to natural contamination [13, 14]. AFB1 has the potential to be convertedinto aflatoxicol or form a complex with albumin in serum. Li et al. [15], resulting in prolonged exposure to the human body [16]. Previous studies have shown that the liver and intestine are the primary organs affected by AFB1 [17].

Mycotoxins, such as AFB1 and AFM1, can potentially induce carcinogenic effects through prolonged and/or significant exposure, leading to the development of malignancies, such as liver cancer and breast cancer. This process is complex and multifactorial. Breast cancer is a common cause of death for women, whereas lung cancer is a leading cause of death for men [6, 18]. In the European Union, the allowable threshold for AFB1 in peanuts and related products is set at 2 μg/kg for direct consumption and 8 μg/kg for indirect consumption. Conversely, the Food and Drug Administration (FDA) in the United States has established a limit of 20 μg/kg for the overall concentration of aflatoxin [17].

The recommendation has been made to estimate cytotoxicity and genotoxicity using IS0 standards 10,993–3 [19] as an essential part of the evaluation process. The performance of cytogenetic tests is desirable for identifying the harmful hazards of specific known materials at various doses [20, 21]. Elderly people have used plants to detect environmental genetic alterations and demonstrate the presence of genotoxic agents [22]. Plant bioassays are preferred because test plants can undergo direct treatment with intricate combinations of environmental contaminants, either within controlled laboratory settings or in their natural habitats [23]. Furthermore, the mutational activity of plant cells is correlated with that of human and animal cells [24].

The cytotoxicity levels of a substance can be determined by observing changes in the mitotic index (MI). Compared with those in the normal group, increased MIs indicate increased cellular division, which can negatively impact cellular function and lead to abnormal cell growth, proliferation and potentially the development of cancerous tumors. Both the suppression and elevation of the MI are important indicators for assessing environmental contamination [21, 25].

The increase in mitotic activity may be caused by chemical changes that regulate cell entry into mitosis [21, 26]. The study also revealed that each treatment resulted in either an increase or a decrease in the mitotic index. A decrease in mitotic activity in plants is a common effect of herbicide efficacy and is influenced by various factors [27].

The mitotic index (MI) is a crucial parameter used to the rate growth is affected by the frequency of cell division. Moreover, the decrease in the mitotic index, as reported by Hartwell [28], depends on whether dividing cells progress to the next cell phase on the basis of the completion of the previous cell phase [28]. This regulatory mechanism is dependent on checkpoints, involve regulatory sites where various genes monitor and control cell states such as cell volume, DNA replication, spindle assembly, and DNA separation [29].

The decrease in the frequencies of metaphase, anaphase, and telophase can be attributed to the action of chemicals on the spindle, resulting in the cessation of division at these stages. Similar findings were obtained from squid pen chitosan on *Vicia faba* [30]. Additionally, [31] reported that chromosome aberration has been used as an index of possible genetic destruction by environmental toxicants in plants for many years and can provide both qualitative and quantitative data on contamination impacts [32].

In the present study**,** compared with control seeds, seeds treated with the studied AFB1 presented increased mitotic abnormalities. These abnormalities were detected in both dividing and non-dividing cells. The cells at the interphase stage presented micronuclei, stickiness, non-congression, chromosome rings, and two groups: micronuclei, star-shaped metaphase, disturbance and oblique at the metaphase stageate separation, diagonal, bridge, laggard, disturbed, and micronucleus were observed at the anaphase stage; and bridge, laggard, diagonal, late separation, micronucleus, and disturbance were observed at the telophase stage [21].

Chromosomal aberrations result from damage to DNA and chromosomal spindle proteins, causing genetic harm [33]. These aberrations can involve changes in chromosomal structure or the total number of chromosomes, which can arise spontaneously or due to exposure to physical or chemical factors [34]. Structural chromosomal abnormalities can be triggered by various conditions, such as DNA breakage, inhibition of DNA synthesis, and replication of abnormal DNA.

Micronuclei at interphase were the first type of chromosomal aberration identified in the present study of non-dividing cells. The presence of certain micronucleirepresents the mutagenic effect under investigation, which is indeed real and serves as a reliable measurement to assess chromosomal aberrations [35]. Many researchers have discussed micronuclei as the most efficient and easiest endpoint for examining the mutagenic effects of chemicals. This can also be attributed to the proper repair of the micronucleus after destruction in the cells of the original parent [35].

Stickiness was the most common type of chromosomal aberration that appeared in metaphase. It covers the entire chromosome complement, making the observation of chromatin masses and the general morphology of chromosomes impossible [36].

Stickiness of the chromatin matrix is often observed during the conventional metaphase stage, possibly due to chromatin dysfunction. Elevated stickiness can also lead to the formation of sticky bridges in both the anaphase and telophase stages, disrupting normal cytokinesis. The presence of sticky chromosomes indicates that pollutants affect the arrangement of chromatin structure. This influence is linked to an imbalance in histone levels or other proteins necessary for proper nuclear chromatin organization [37]. Furthermore, these stickiness abnormalities may be classified as spindle-related abrrations, which are believed to be induced by cytotoxicity. There was a significant increase in clastogenicity following the treatment [21, 38].

Nondisjunction occurs when one or two chromosomes are not properly aligned in the cytoplasm, potentially due to the presence of micronuclei in the mitotic cycle. This phenomenon is a reliable indicator of chromosomal abnormalities in the *Vicia faba* assay [21, 35].

Chromosomal rings observed during metaphase typically result from two terminal breaks in both arms of the chromosome, which then fuse together at the broken ends. Alternatively, chromosomal rings can form from the fusion of a broken chromosome end with the telomere region of another chromosome, leading to genetic material loss. In another scenario, these structures canform through the fusion of sub-telomeric sequences or telomeres without genetic material loss, resulting in intact ring chromosomes [39, 40].

isrupted metaphase can be caused by partial disruption of the spindle machinery or inhibition of spindle formation due to the presence of zinc ions [41, 42]. Additionally, uneven chromosome distribution in the spinel apparatus may indicate this **[**abnormality, possibly due to partial suppression of spindle creation or the loss of microtubules in spindle fibers] [43, 44].

Other abnormalities observed during various mitotic stages, such as diagonal deviation disturbances, star metaphase, and free chromosome abnormalities, are associated with spindle inhibition [45]. Diagonal deviations werenoted during anaphase and telophase in the root tip cells of *Vicia faba*. In cases of aberrations, the two groups of chromosomes within a cell are not aligned along a common axis. This misalignment can be attributed to impaction within the spindle mechanism [42].

Chromosomal bridges may result from chromosome breakdown and subsequent proximal chromatid recombination. This process leads to chromosomes that are dysenteric and formbridges [32, 46]. The formation of bridges in chromosomes can be understood by considering the overall adhesive properties of chromosomes and the subsequent prevention of anaphase separation [47].

A remarkable correlation exists between stickiness and bridges. This finding supports the hypothesis that the occurrence of bridges, whether chromosomal or chromatidal, is most likely due to general stickiness rather than chromosome cleavage and reunion [36].

Lagging chromosomes can be attributed to a hindrance in the proper organization and functioning of the spindle apparatus rather than a complete failure of the spindle fibers. This can result in the uneven alignment of chromosomes [48]. This phenomenon may be caused by a delayed termination ofchromosome ends or a deficiency in chromosomal movement, which is associated with the spindle mechanism [49]. Late separation or a delay in theseparation process may lead to illness, as indicated by previous studies [21, 50].

Electrophoretic techniques for protein analysis have been established as appropriate approaches for assessing the potential mutagenic impacts resulting from persistent and cumulative pollution. These techniques allow for the correlation of observed variations with chromosomal abnormalities induced by environmental pollutants [51, 52]. Protein electrophoresis is a viable method for the identification of individual polypeptides because the analyzed proteins are direct structural manifestations of gene loci and therefore serve as accurate representations of the prevailing genome configuration [53].

Several researchers have successfully used electrophoretic SDS‒PAGE to establish the biochemical genetic fingerprints of many plant species [54]. Moreover, the use of electrophoretic analysis to study proteins provides valuable insights into the structural genes and regulatory mechanisms that govern the biosynthesis pathways associated with these proteins [55].

SDS‒polyacrylamide gel electrophoresis (SDS‒PAGE) is a discontinuous gel method that has been widely used in various scientific studies. It is known for its speed and high accuracy, allowing the separation of proteins into segments on the basis of their size, which results in the generation of bands with varying molecular weights across gels [56] and [57]. SDS‒PAGE has been utilized in many previous studies to evaluate how environmental stress affects protein profiles [57, 58].

Each protein band observed in the protein profile of an organism can be attributed to a distinct transcriptional event [59]. Furthermore, the emergence of a novel band can be elucidated by a mutational occurrence inside the regulatory system of a previously unexpressed gene, which matches [60]. However, the absence of some bands in the protein profile of the root tip cells of *Vicia faba* after treatment with the studied AFB1 could be explained by the absence or inactivation of the genes associated with them [61, 62]. Additionally, the changes in protein patterns after exposure to various chemical substances, as noted by many authors [63, 64], and the presence or absence of protein bands can be utilized as diagnostic parameters for a group or certain taxon [65, 66].

Estimating genetic damage in plants at the level of deoxyribonucleic acid is preferable because of its inherent sensitivity and limited response time [67]. DNA fingerprinting offers a variety of reliable diagnostic assays that can be used for evaluating genotoxicity [58, 68, 69].

Both the ISSR and RAPD markers were used as molecular indicators to study the impact of different concentrations of AFB1 at various exposure times. The RAPD technique provides a quick and effective way to screen DNA sequence-based polymorphisms across multiple loci. One significant advantage of RAPD is its ability to eliminate the need for DNA presequencing [70]. These findings highlighted the effectiveness of RAPD analysis in distinguishing between the different subjects studied in *Vicia faba* treated with varying concentrations, as mentioned previously [71], which proved the simplicity of RAPD assays for determining genetic polymorphisms.

The ISSR technique is widely recognized as one of the simplest and most commonly employed markers in the field of polymerase chain reaction, making ita widely used molecular technique [72]. Intersimple sequence repeats are DNA-based markers that investigate genetic variations, specifically polymorphisms, within intermicrosatellite loci [73]. The identification ofmutational outcomes of various metallic elements and the use of a reliable technique for genotoxicity assays are highly important [74–76].

The ISSR indicator is a useful technique for estimating the impact of silver nanoparticles on DNA levels [22, 77]. The ability of AgNPs to induce DNA damage and possibly cause cell death was previously demonstrated [78]. In this study, an analysis of ISSR banding profiles revealed polymorphism exposure to various concentrations of AFB1.

he presence of new bands was thought to be associated with genetic changes, whereas the absence of bands could indicate DNA alterations [79].

Disruptions in the stability of the genomic template can occur as a result of various alterations at the DNA level, such as DNA damage and impairments in DNA repair and replication systems. A qualitative assessment of the genotoxic effects on genomic template stability can be performed [58, 80]. The evaluation of genomic template stability (GTS) has been employed to examine various forms of DNA damage and subsequent mutations in mammals, plants, and bacterial cells [81, 82]. The genotoxicity of silver nanoparticles was calculated via a genotoxicity testing system (GTS) as a measurement tool [57, 83] and for zinc nanoparticles [58, 84]. The GTS refers to a qualitative assessment of the modifications detected via the SDS‒PAGE and ISSR techniques resulting from exposure to silver nanoparticles compared with the profiles obtained from the control sample. The observations of Çekiç et al. [77] indicate that the observed reduction in GTS may be attributed to elevated levels of oxidative stress, which is dependent upon the concentration at which DNA destruction is detected [85].

According to a study conducted by Alm-Eldeen et al. 2017 [86], mice exposed to AFB1 presented significant upregulation of the p53 protein, a proapoptotic indicator, and downregulation of the bcl2 protein, an antiapoptotic marker, in hepatic tissues [86]. The available literaturesuggests that AFB1 can cause DNA malformation, suppress the expression of p27, and promote cell apoptosis in cultured liver cells [87]. AFB1 can also increase the expression levels of the p53 and Bax proteins, both of which are proapoptotic proteins in hepatic cells [88].

Rotimi et al. 2016 [89] reported that feeding rats low-protein diets supplemented with AFB1 led to genomic modifications that correlated with significant increases in p53 expression and caspase-3 activity [89]. A previous study [90] also reported increased expression of Bax, caspase-3, and proapoptotic p53 along with decreased levels of the antiapoptotic protein Bcl-2. [90]. The p53 pathway, which is responsible for the toxicityby AFB1, is an apoptotic process influenced by external factors [91]. AFB1-induced oxidative stress is widely recognized as a key factor contributing to apoptosis. Exposure to AFB1 and OTA results in a marked decrease in cell viability, and the extent of this decrease is dependent on the dosage administered. the same experimental conditions, the presence of these mycotoxins led to increased levels of fragmented DNA [92]. Furthermore, p53 is activated by DNA degradation, leading to a significant decrease in the expression of bcl-2, an antiapoptotic protein. On the basis of these data, both OTA and AFB1 may be involved in cell death mechanisms.

It is hypothesized that the process of chromatin condensation arises from a series of recurrent events activated by the suppression of messenger RNA production following exposure to the chemical carcinogen AFB1. The absence of messenger RNA synthesis results in a reduction in protein synthesis, subsequently inducing the dephosphorylation of histone H1 and initiating the compaction of chromatin in living organisms, as supported by [93]. Moreover, HepG2 cells were subjected to varying doses of AFB1 according to the manufacturer’s instructions. Ultra structural analysis revealed that, when cultivated with AFB1, HepG2 cells exhibited various cellular changes. These changes included the destruction of mitochondria, condensation of the nucleus, and disruption of cell connections. Specifically, malfunctioning gap junctions were observed, leading to a compromised ability of cells to communicate. At the genomic level, AFB1 leads to the formation of AFB1-N7-guanine adducts, resulting in the death of apoptotic cells and the suppression of p53 protein production [94].

In the group exposed to AFB1 alone, the protein levels of Bax and the cleaved form of poly (ADP‒ribose) polymerase (PARP) increased, whereas the level of Bcl-2 decreased [95]. Furthermore, [96] the activity of certain nuclear enzymes involved in DNA repair was examined after the injection of AFB1 in rats. This study revealed a significant increase in the expression of poly (ADP‒ribose) polymerase, DNA polymerase beta, and DNA ligase in rats exposed to AFB1.

The results of image cytometric measurements revealed a correlation between the dose of AFB1 (AFB1) and its impact on various components of the mitotic cell phases in the root tip cells of *Vicia faba* L. The primary observable outcome was increased number of cells in the G0/G1 phase, leading to a reduction in the number of cellsprogressing through the S phase, G2 phase, and M phase of the cell cycle. It was also suggested that the identified toxin functions as a suppressor of cell cycle progression, specifically at the G1 checkpoint. The observed decrease in seedling growth can be attributed to the suppression of mitotic activity caused by AFB2 treatment, as supported by previous studies [97].

## Materials and Methods4.1.Chemicals

The chemicals and reagents used in the present study, their CAS-Number, purity and suppliers are given in Table 6.

**Table 6:** The chemicals and reagents used in the current study

Chemical	CAS-Number	Suppliers
Aflatoxin B1	1162–65-8	Sigma-Aldrich
Carbol Fuchsin	HT8018	Sigma-Aldrich
Tris–HCl	1185_531	Sisco Research Laboratories Pvt. Ltd
Ehylenediaminetetraacetic acid (EDTA)	6381–92-6	AG scientific
Phenylmethylsulfonyl fluoride (PMSF)	329–98-6	Sigma-Aldrich
A polyacrylamide	738,743-1G	Sigma-Aldrich
A DNeasy Plant Mini Kit	69,104	QIAGEN
Agarose	MB755-0100	Bio-Helix

### Evaluation of the cytological impacts of the studied aflatoxin B1 (AFB1) via the *Vicia faba* plant assay

Broad bean(*Vicia faba*) seeds (2n = 12) (Giza 3) were purchased from the Agriculture Research Center, Crop Research Section, Giza, Egypt. Healthy seeds of the same size from the same stock were left in 200 ml of tap water at 25 °C for 24 h and then placed within 2 sheets of damp cotton. When the roots appeared to be 1–1.5 cm in length, they were removed. One concentration of each aflatoxin B1 (AFB1) (control, 5 ng, 67 ng, 181 ng, and 236 ng) was applied for three different exposure times (3, 6, and 12 h). A dual-staining technique that integrates the modified Carbol Fuchsin reaction, as described by Koa in 1975 [98, 99], was employed.

The slides were observed via an Olympus CX40 electric microscope, specifically with a 100 × objective lens in conjunction with oil immersion. A minimum of 2000 cells were analyzed from approximately 10 slides for each treated sample to detect the rate of mitotic division and phase index and to score the types and frequencies of chromosomal abnormalities. The mitotic index (MI) was assessed to determine the rate of cell division during mitosis. The dividing cells were examined to determine the types and incidences of mitotic and chromosomal aberrations produced by various AFB1 treatments. The frequency of abnormalities in each stage of division was calculated. The most representative cell with aberrations for each abnormality was taken by an Olympus camera (SC 35 type 12 model).

The various stages of mitosis were scored, and chromosomal aberrations were calculated as the mitotic index, phase index, and total abnormality percentage at different phases of cell division. Mitotic index (MI), phase index (PI), and total abnormality percentage (TAP).

### SDS‒PAGE electrophoresis

The extraction buffer utilized in this experiment consisted of approximately 20 mM Tris–HCl at a pH of 8.0. Additionally, the buffer contained two mM ethylenediaminetetraacetic acid (EDTA) and one mM phenylmethylsulfonyl fluoride (PMSF). Dry seeds of the faba bean cultivar Giza3 were subjected to different AFB1 treatments for three exposure times: 3, 6, and 12 h. The protein obtained from the leaves was subjected to SDS‒PAGE on a 15% polyacrylamide gel following the methodology described by Laemmli 1970 [100] and subsequently changed to [101]. The protein electrophoretic profile of the leaves was assessed by quantifying the presence (1) or absence (0) of a band with a certain molecular mass. The protein profile was assessed via the Bio-Rad Gel Documentation System, specifically the Bio-Rad-Gel-Doc Model 2000.

### Molecular analysis

DNA isolation from plant leaf samples was performed via a DNeasy Plant Mini Kit (QIAGEN). In this investigation, five primers for both RAPD and ISSR and their sequences were used as illustrated in Table 7. DNA amplification was performed in 30-µl vials according to [102] with some modifications. DNA amplification was performed with an automated thermal cycle device, specifically the Techno 512 model. The samples were subjected to gel electrophoresis via a 1.5% agarose gel. The data for each treatment were scored as (1) to indicate existence and (0) to indicate disappearance.

**Table 7:** Name and Sequences of ISSR and RAPD primers

Molecular Markers	Primer Name	Sequence( 5´- 3`)
**ISSR**	ISSR 1	(CT)8G
	ISSR 2	(AG)8G
	ISSR 3	(AG)8 C
	ISSR 4	(GA)8 T
	ISSR 5	(AG)8TG
**RAPD**	OPA2	TGCCGAGCTG
	OPA13	CAGCACCCAC
	OPE3	CCAGATGCAC
	OPH13	GACGCCACAC
	OPA11	CAATCGCCGT

### Genomic template stability

The calculation of genomic template stability (GTS%) was performed via the equation shown below. The formula for GTS is expressed as follows: GTS = (I—a/n) × 100, where I represents the average number of polymorphic bands discovered in each treated sample and n denotes the total number of bands observed in the control. The percentage of polymorphisms found in the SDS‒PAGE, ISSR, and RAPD profiles was determined by considering the disappearance of normal bands, the introduction of new bands, or changes in band intensities compared with the control profile. The proposition posits that modifications in DNA profiles resulting from exposure to genotoxic agents can be interpreted as variations in genomic template stability [58, 103].

#### Flow cytometric analysis

*Vicia faba* seeds were exposed to different AFB1 treatments for three exposure times (3, 6, and 12 h) and then used to prepare flow cytometric samples. Cell analysis was performed via an Accuri C6 Plus flow cytometer manufactured by Becton Dickinson and located at Sunnyvale, California, United States. This device was used for flow cytometry analysis. At the Mansoura Research Center for Cord Stem Cells (MARC-CSs), Faculty of Medicine, Mansoura University, Egypt.

### Statistical analysis

MS Office 2010 was utilized for data entry and processing. The cytotoxic potential was examined by assessing the mitotic index (MI), phase indices (PI), and the overall percentage of abnormalities during various stages of cell division. The data underwent statistical analysis through t-tests to assess the differences between various treatments and the untreated sample [104].

## Conclusion

ISSR markers have been shown to be more reliable than RAPD markers for detecting polymorphisms. The ISSR technique has several advantages, such as the ability to withstand high annealing temperatures and repetitive amplification, making it a cost-effective method. This herb has been widely used with medicinal plant species and may be a useful tool for DNA fingerprinting. Additionally, the inter simple sequence repeat (ISSR) technique has been utilized to assess genetic diversity within different plant species.

## Data Availability

All recorded, measured, and analyzed datasets (e.g., independent measurements of the three biological and technical replicates) were completely incorporated within the manuscript's main text and its accompanying data files.
